# Structural and Photophysical Trends in Rhenium(I) Carbonyl Complexes with 2,2′:6′,2″-Terpyridines

**DOI:** 10.3390/molecules29071631

**Published:** 2024-04-05

**Authors:** Joanna Palion-Gazda, Katarzyna Choroba, Anna Maria Maroń, Ewa Malicka, Barbara Machura

**Affiliations:** Institute of Chemistry, University of Silesia, 9 Szkolna Str., 40-006 Katowice, Poland; katarzyna.choroba@us.edu.pl (K.C.); anna.maron@us.edu.pl (A.M.M.); ewa.malicka@us.edu.pl (E.M.)

**Keywords:** Re(I) carbonyl complexes, 2,2′:6′,2″-terpyridine-based ligands, ground- and excited-state properties, structure–property relationships

## Abstract

This is the first comprehensive review of rhenium(I) carbonyl complexes with 2,2′:6′,2″-terpyridine-based ligands (R-terpy)—encompassing their synthesis, molecular features, photophysical behavior, and potential applications. Particular attention has been devoted to demonstrating how the coordination mode of 2,2′:6′,2″-terpyridine (terpy-κ^2^N and terpy-κ^3^N), structural modifications of terpy framework (R), and the nature of ancillary ligands (X—mono-negative anion, L—neutral ligand) may tune the photophysical behavior of Re(I) complexes [Re(X/L)(CO)_3_(R-terpy-κ^2^N)]^0/+^ and [Re(X/L)(CO)_2_(R-terpy-κ^3^N)]^0/+^. Our discussion also includes homo- and heteronuclear multicomponent systems with {Re(CO)_3_(R-terpy-κ^2^N)} and {Re(CO)_2_(R-terpy-κ^3^N)} motifs. The presented structure–property relationships are of high importance for controlling the photoinduced processes in these systems and making further progress in the development of more efficient Re-based luminophores, photosensitizers, and photocatalysts for modern technologies.

## 1. Introduction

Rhenium(I) tricarbonyl coordination compounds with phenanthroline-based ligands were the first metal carbonyls reported to exhibit luminescence in solution at room temperature (RT). The broad and non-structured emission band of these systems was attributed to phosphorescence from the triplet excited state of metal-to-ligand charge transfer (MLCT) nature, evolving from the optically populated ^1^MLCT state via intersystem crossing (ISC) due to the large spin–orbit coupling constant of the Re(I) ion [1]. Since Wrighton’s pioneering report, photoluminescent tricarbonyl rhenium(I) compounds with ligands based on diimine cores have been widely synthesized and extensively explored in order to: (i) understand light-induced energy and charge transfer processes in transition metal complexes, (ii) design functional materials suitable for use in optoelectronics, photoinduced catalysis, and biomedicine, as well as (iii) establish reliable relationships between structural modifications of diimine (N^∩^N)/ancillary (X/L) ligands and the photophysical behavior of the resulting [Re(X/L)(CO)_3_(N^∩^N)]^0/+^, necessary for progress in improving phosphorescent materials and potent chemotherapeutic drugs. It has been evidenced that [Re(X/L)(CO)_3_(N^∩^N)]^0/+^ complexes may be characterized by different triplet excited states, including metal-to-ligand charge transfer (MLCT), ligand-to-ligand-charge-transfer (LLCT), intraligand (IL), intraligand-charge-transfer (ILCT), or their superposition, and each of these excited states brings characteristic photophysical properties to the resulting complex [2,3,4,5,6,7,8,9,10,11,12,13]. Due to the rich and finely tuned photochemistry, achieved through an appropriate combination of diimine and ancillary ligands, accompanied by their efficient synthesis, and possessing good thermal and photochemical stability, the systems [Re(X/L)(CO)_3_(N^∩^N)]^0/+^ have proven to be appealing for various applications. These include photocatalytic carbon dioxide reduction [14,15,16,17,18,19] and H_2_ evolution [20,21,22,23], organic light-emitting diodes (OLEDs) [24,25,26,27], phosphorescent molecular sensing [28,29], and medicinal applications [11,13,30,31].

Within this work, we present a comprehensive review of Re(I) carbonyl complexes with 2,2′:6′,2″-terpyridine-based ligands (R-terpy). The rhenium(I) carbonyl complex with 2,2′:6′,2″-terpyridine (terpy) was first reported in 1988 by Juris et al. In analogy to diimine tricarbonyl Re(I) coordination compounds, it was obtained by reacting [Re(CO)_5_Cl] with the terpy ligand in toluene under reflux conditions. Initially identified as [ReCl(CO)_2_(terpy-κ^3^N)], with terpy *meridionally*-coordinated to the Re(I) center (terpy-κ^3^N) [32], the more advanced spectroscopic investigations and isolation of the terpy Re(I) carbonyl complex in a monocrystalline form, followed by subsequent X-ray analysis, revealed a bidentate coordination mode of terpy (terpy-κ^2^N) in the product of the reaction [33,34,35]. As widely reported later [36,37,38], further removal of the carbonyl group and formation of [ReCl(CO)_2_(terpy-κ^3^N)] require much harsher reaction conditions. The Re(I) carbonyls with *meridionally* coordinated terpy are obtained by the solid-state thermal elimination of CO following the coordination of the pendant pyridyl group in the [ReCl(CO)_3_(terpy-κ^2^N)] precursor [36,37,38].

Regarding photophysical behavior, [ReCl(CO)_3_(terpy-κ^2^N)] was initially found to be non-emissive in solution at RT [32], which led to a noticeable decline in scientific interest in this class of compounds compared to diimine Re(I) tricarbonyl compounds. The striking difference in the emission properties between [ReCl(CO)_3_(terpy-κ^2^N)] and its analog [ReCl(CO)_3_(bipy)] (bipy—2,2′-bipyridine) was rationalized by the thermal coupling of ^3^MLCT and ^3^IL excited states, diminished in the bipy-based Re(I) carbonyl due to the larger energy separation between ^3^MLCT and ^3^IL [39]. However, the repetition of spectroscopic investigation of [ReCl(CO)_3_(terpy-κ^2^N)] by Amoroso et al. showed that the complex is weakly emissive, both in solution and the solid state [39]. Since 2013, there has been renewed interest in the photophysics of terpy-based Re(I) carbonyl complexes. It was assumed that variations in the terpy core and ancillary ligand (X/L) might lead to a significant enhancement of photoluminescence and improve the photocatalytic performance of [Re(X/L)(CO)_3_(R-terpy-κ^2^N)]^0/+^ systems, offering a chance to develop new functional materials for modern technologies and expand the fundamental knowledge and understanding in optimizing the photophysical properties of transition metal complexes. The great advantage of 2,2′:6′,2″-terpyridines is their efficient synthesis method (Kröhnke condensation), allowing for the incorporation of a wide range of electron-withdrawing or donating groups into the terpy core. Furthermore, compared to diimine derivatives, 2,2′:6′,2″-terpyridine-based ligands provide the possibility of additional modification of the photophysical properties of Re(I) carbonyl systems through coordination to the Re(I) center.

The purpose of this review is to demonstrate the effect of the 2,2′:6′,2″-terpyridine coordination mode (terpy-κ^2^N and terpy-κ^2^N), structural modifications of the terpy core (R-terpy), and the nature of ancillary ligands (X/L) in controlling the photophysical properties of mononuclear and multicomponent systems with {Re(CO)_3_(R-terpy-κ^2^N)} and {Re(CO)_2_(R-terpy-κ^3^N)} motifs.

## 2. Structural Features of [ReX(CO)_3_(terpy-κ^2^N)] and [ReX(CO)_2_(terpy-κ^3^N)] Systems and Their Derivatives

**Figure 1:**
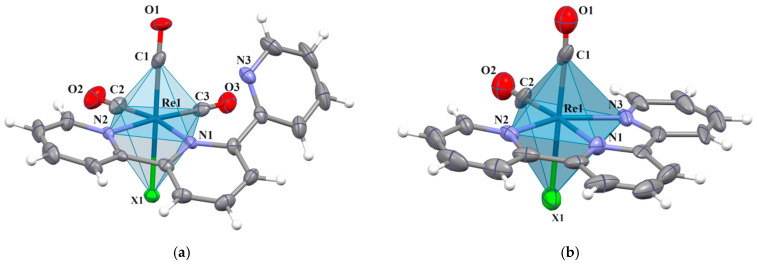
Perspective views of [ReX(CO)_3_(terpy-κ^2^N)] (**a**) and [ReX(CO)_2_(terpy-κ^3^N)] (**b**) molecular structures indicating the numbering schemes used in Table 1 and Tables S1–S3.

The solid-state structures of [ReX(CO)_3_(terpy-κ^2^N)] and [ReX(CO)_2_(terpy-κ^3^N)] (X = Cl, Br) were reported in [33,34,35,38], and the important structural features of these systems are depicted in Figure 1 and summarized in Table 1. In both [ReX(CO)_3_(terpy-κ^2^N)] and [ReX(CO)_2_(terpy-κ^3^N)], the Re(I) atom adopts a distorted octahedral geometry. The coordination sphere of [ReX(CO)_3_(terpy-κ^2^N)] is defined by three carbonyl ligands in a *facial* arrangement, a chloride ion, and two nitrogen atoms of the bidentate-coordinated terpy ligand (Figure 1a). On the other hand, the geometry around the Re(I) center in [ReX(CO)_2_(terpy-κ^3^N)] is determined by two *cis*-oriented carbonyl groups, a chloride ion and three nitrogen atoms of a *meridionally*-coordinated terpy ligand (Figure 1b). From a structural point of view, [ReX(CO)_3_(terpy-κ^2^N)] can be considered as a derivative of the diimine tricarbonyl complex [ReX(CO)_3_(bipy)] with an uncoordinated pyridyl ring as a substituent.

A different coordination mode of terpy induces noticeable variations in the bond lengths and angles in [ReX(CO)_3_(terpy-κ^2^N)] and [ReX(CO)_2_(terpy-κ^3^N)] (Table 1). The Re–N distances, especially those between the metal ion and the central pyridine ring of terpy, undergo shortening after conversion from the κ^2^N to κ^3^N coordination mode, indicating a stronger Re-terpy interaction in the complex bearing a *meridionally*-coordinated terpy ligand. In turn, the Re–CO bonds become elongated upon changing the terpy coordination mode from bidentate to tridentate, and elongation of the Re–CO distances in [ReX(CO)_2_(terpy-κ^3^N)] is accompanied by the shortening of C–O bond lengths relative to those in [ReX(CO)_3_(terpy-κ^2^N)]. Opposite trends are also noticed when Re–N_central pyridine_ and Re–N_lateral pyridine_ distances are compared for [ReX(CO)_3_(terpy-κ^2^N)] and [ReX(CO)_2_(terpy-κ^3^N)]. While the Re(I) complexes with terpy-κ^3^N show Re–N_central pyridine_ bond lengths significantly shorter than Re–N_lateral pyridine_ ones, the Re–N_central pyridine_ distances are noticeably elongated in relation to Re–N_lateral pyridine_ ones in the terpy-κ^2^N systems. Expectedly, the longer Re–N bond distances induce the smaller bite angles in [ReX(CO)_3_(terpy-κ^2^N)] relative to those for [ReX(CO)_2_(terpy-κ^3^N)] [40]. For both systems, [ReX(CO)_3_(terpy-κ^2^N)] and [ReX(CO)_2_(terpy-κ^3^N)], however, due to the geometrical constraints imposed by the formation of the five-member chelate rings upon coordination of terpy, the angles N–Re–N are noticeably smaller than 90°, leading to an essential distortion from the ideal octahedron. In Re(I) complexes with the terpy ligand coordinated in a bidentate mode, the relevant angular distortion of the coordination sphere is also contributed by steric interactions between the uncoordinated peripheral pyridine ring and the carbonyl group C(3)–O(3), leading to the loss of the coplanarity of the terpy skeleton in [ReX(CO)_3_(terpy-κ^2^N)] and enlargement of the C(3)–Re(1)–N(1) bond angle. For reported structures of [ReX(CO)_3_(terpy-κ^2^N)], the bond angles C(3)–Re(1)–N(1) are the largest ones among those for *cis*-arranged ligands, falling in the range 100.7(4)–102.67(3)°. The dihedral angles between the least squares planes of the central pyridine and the pendant pyridyl group in [ReX(CO)_3_(terpy-κ^2^N)] structures vary from 40.2(4) to 69.58(3)° (Table 1).

**Scheme 1:**
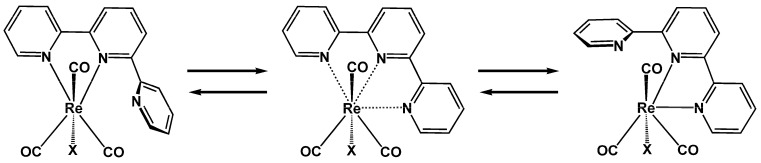
The interconverting structures of the complex [ReX(CO)_3_(terpy-κ^2^N)] in solution.

As widely reported in [33,35,41,42,43,44], the terpy bidentate coordination mode of [ReX(CO)_3_(terpy-κ^2^N)] is also maintained in solution, with the terpy ligand exhibiting fluxionality. It switches its metal coordination sites between pendant pyridyl groups via an associative mechanism implying a seven-coordinate intermediate (Scheme 1).

**Table 1:** Selected bond lengths (Å) and angles (s) for [ReX(CO)_3_(terpy-κ^2^N)] and [ReX(CO)_2_(terpy-κ^3^N)] (X = Cl, Br).

	[ReX(CO)_3_(terpy-κ^2^N)]								[ReCl(CO)_3_(terpy-κ^3^N)]
Refcode *	WAFVOO [33]	WAFVOO1 [33]	BOFYOL	ILEGIR [38]	PAVKUS [34]	SUHDII [35]	SUHDII01 [35]	SUHDII02 [35]	ILEHIS [38]
X	Cl	Cl	Cl	Cl	Cl	Br	Br	Br	–
Bond lengths									
Re(1)–X(1)	2.4877(6)	2.4936(7)	2.4907(8)	2.496(2)	2.4932(11)	2.6306(13)	2.6409(3)	2.6408(6)	2.489(2)
Re(1)–N(1)	2.2059(5)	2.214(2)	2.215(2)	2.232(9)	2.228(3)	2.210(8)	2.2283(19)	2.233(3)	2.079(7)
Re(1)–N(2)	2.1710(3)	2.161(2)	2.159(3)	2.165(6)	2.151(2)	2.144(8)	2.174(3)	2.173(4)	2.118(8)
Re(1)–N(3)									2.124(8)
Re(1)–C(1)	1.9019(5)	1.907(3)	1.915(3)	1.902(10)	1.880(4)	1.896(13)	1.886(3)	1.895(4)	1.963(8)
Re(1)–C(2)	1.9085(4)	1.909(3)	1.904(3)	1.892(11)	1.903(4)	1.889(11)	1.913(3)	1.911(4)	1.917(8)
Re(1)–C(3)	1.9363(3)	1.928(2)	1.937(3)	1.935(8)	1.909(4)	1.898(11)	1.922(3)	1.921(5)	
C(1)–O(1)	1.1509(3)	1.154(4)	1.146(4)	1.150(12)	1.157(6)	1.158(15)	1.154(3)	1.152(5)	1.061(11)
C(2)–O(2)	1.1349(3)	1.150(3)	1.158(4)	1.159(14)	1.153(5)	1.158(14)	1.150(3)	1.150(5)	1.14(1)
C(3)–O(3)	1.1139(2)	1.147(3)	1.146(4)	1.125(11)	1.171(5)	1.160(13)	1.152(4)	1.154(7)	
Bond angles									
N(1)–Re(1)–Cl(1)	83.21(3)	83.16(6)	82.66(7)	81.59(19)	81.98(8)	82.3(2)	82.05(5)	82.09(9)	82.04(19)
N(2)–Re(1)–Cl(1)	83.62(2)	83.35(6)	82.26(7)	84.05(18)	82.66(9)	84.6(2)	85.71(5)	85.68(8)	85.29(19)
N(3)–Re(1)–Cl(1)									88.71(19)
N(1)–Re(1)–N(2)	75.15(1)	74.74(7)	75.02(9)	74.50(20)	74.3(1)	74.2(3)	74.80(8)	74.63(11)	77.3(3)
N(1)–Re(1)–N(3)									76.6(3)
N(2)–Re(1)–N(3)									153.8(3)
C(1)–Re(1)–Cl(1)	178.046(1)	178.22(8)	177.53(11)	179.9(3)	175.68(12)	176.8(3)	177.89(9)	177.61(13)	176.3(2)
C(1)–Re(1)–N(1)	94.64(3)	96.66(10)	95.51(12)	98.3(4)	99.41(14)	98.8(4)	96.37(9)	95.92(14)	94.5(3)
C(1)–Re(1)–N(2)	94.45(2)	94.89(9)	98.90(11)	95.9(3)	93.75(15)	98.6(4)	92.53(11)	92.53(15)	92.7(3)
C(1)–Re(1)–N(3)									91.8(4)
C(2)–Re(1)–Cl(1)	90.78(3)	90.42(9)	96.05(9)	91.7(3)	89.71(14)	92.3(4)	92.70(8)	92.68(12)	91.8(2)
C(2)–Re(1)–N(1)	169.974(3)	169.58(10)	171.13(11)	169.2(3)	168.48(14)	170.7(4)	171.26(12)	171.12(16)	173.6(3)
C(2)–Re(1)–N(2)	96.26(1)	96.43(9)	96.11(11)	95.5(3)	96.82(14)	97.9(4)	97.92(11)	97.91(14)	103.9(3)
C(2)–Re(1)–N(3)									101.8(3)
C(2)–Re(1)–C(1)	89.09(3)	89.50(12)	86.01(14)	88.5(4)	88.33(18)	87.0(5)	88.70(11)	89.12(16)	91.7(4)
C(2)–Re(1)–C(3)	85.34(2)	85.68(11)	86.70(14)	87.5(4)	85.87(17)	86.6(5)	86.11(13)	85.75(17)	
C(3)–Re(1)–Cl(1)	90.59(2)	91.43(9)	89.22(9)	92.9(3)	94.52(14)	88.1(4)	91.58(8)	91.63(12)	
C(3)–Re(1)–N(1)	102.67(1)	102.62(9)	102.04(12)	101.1(4)	102.64(14)	100.7(4)	100.95(10)	101.50(14)	
C(3)–Re(1)–N(2)	174.003(1)	174.38(10)	171.26(10)	175.0(4)	176.07(16)	171.6(4)	175.23(9)	175.55(13)	
C(3)–Re(1)–C(1)	91.35(2)	90.33(11)	89.53(13)	87.1(4)	89.17(18)	88.7(5)	90.09(13)	90.06(18)	
Dihedral angle **	69.58(3)	68.95(9)	63.75(11)	40.2(4)	53.27(13)	52.9(4)	42.31(9)	42.26(13)	–

* Refcode in the Cambridge Structural Database, version 2023.3 (November 2023) [45]. ** Dihedral angle between the least squares planes of the central pyridine and pendant pyridyl group.

Analysis of X-ray results for [ReX(CO)_3_(R-terpy-κ^2^N)] and [ReX(CO)_2_(R-terpy-κ^3^N)] bearing 4′-substituted 2,2′:6′,2″-terpyridines (R-terpy) indicates that the introduction of different types of groups into the terpy core, directly or via an aromatic linker, does not induce noticeable structural variations in the {ReClN_2_C_3_} coordination core. The bond lengths and bond angles around the Re(I) ion are comparable to those for the model chromophores [ReX(CO)_3_(terpy-κ^2^N)] and [ReX(CO)_2_(terpy-κ^3^N)] (Tables S1 and S2). Also, the replacement of the halide ion by a neutral ligand and formation of [ReL(CO)_3_(R-terpy-κ^2^N)]^+^ and [ReL(CO)_2_(R-terpy-κ^3^N)]^+^ does not generate large changes in structural parameters in the {ReN_2_C_3_} {ReN_3_C_2_} coordination units, respectively (Tables S2 and S3). Among complexes [Re(X/L)(CO)_3_(R-terpy-κ^2^N)]^0/+^, structural differences are mainly noticed regarding the relative orientation of the appended aromatic group and central pyridine ring as well as the dihedral angle between the least squares planes of the central pyridine and the uncoordinated pyridyl group.

## 3. Photophysical Properties of [ReX(CO)_3_(terpy-κ^2^N)] and [ReX(CO)_2_(terpy-κ^3^N)]

**Figure 2:**
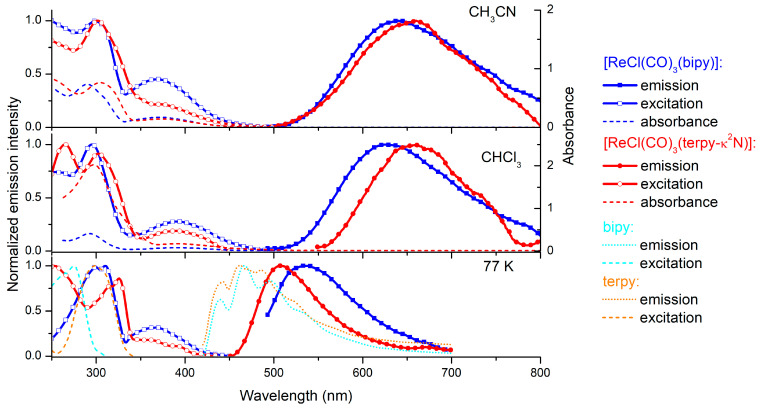
Comparative analysis of the absorption and emission properties of [ReCl(CO)_3_(terpy-κ^2^N)] and [ReCl(CO)_3_(bipy)], performed using the spectral data reported in our previous works [46,50].

The optical properties of [ReX(CO)_3_(terpy-κ^2^N)] (X = Cl, Br) have been extensively investigated, as reported in [32,39,46,47,48,49,50]. The absorption behavior of [ReX(CO)_3_(terpy-κ^2^N)] was found to be typical of [ReX(CO)_3_(diimine)] chromophores. Similar to the structurally related [ReX(CO)_3_(bipy)], the UV-Vis spectra of [ReX(CO)_3_(terpy-κ^2^N)] display intense bands below 350 nm attributed to π→π* and n→π* intraligand (IL) transitions, along with moderate, broad absorption in the range of 350–450 nm largely assigned to MLCT excitations (Figure 2 and Table S4). This assignment was also supported by density functional (DFT) calculations [46,47,48].

Regarding the photoluminescence properties of the Re(I) complex bearing the terpy-κ^2^N ligand, there is a significant discrepancy in the literature data (Table 2). The complex [ReCl(CO)_3_(terpy-κ^2^N)] was initially reported as non-luminescent in DMF solution at RT, displaying emission only at 77 K in a 9:1 DMF–CH_2_Cl_2_ glass [32]. Subsequent studies of [ReCl(CO)_3_(terpy-κ^2^N)] [39] revealed weak emission in its acetonitrile solution at 506 nm following the photoexcitation at 360 nm, but no photoluminescence quantum yield and lifetimes were recorded. The authors of [49] demonstrated that [ReCl(CO)_3_(terpy-κ^2^N)] in CH_2_Cl_2_, excited at 442 nm, displayed the emission band at 509 nm, with photoluminescence quantum yield and decay times recorded as 0.3 % and 2.02 μs, respectively. Worthy of note, the emission spectrum of [ReCl(CO)_3_(terpy-κ^2^N)], presented in [49], comprised two emission bands (at 509 and ~630 nm), but only the higher energy one was discussed by the authors. Concerning time-resolved measurements, the accuracy cannot be estimated based on the decay curves included in the ESI materials [49]. The latest studies conducted by our research group [46] revealed somewhat different photophysics of [ReCl(CO)_3_(terpy-κ^2^N)]. The emission of this complex was found to occur above 600 nm, namely at 638 nm in CHCl_3_ and 656 nm in MeCN, with excited-state lifetimes falling in the nanosecond range (Figure 2 and Table 2). These emission maxima and lifetimes correlate well with the results for the related bromide complex [ReBr(CO)_3_(terpy-κ^2^N)] in acetonitrile, recently reported in [47]. According to the findings of the latest research [46,47], the Re(I) complexes with the bidentate terpy ligand exhibit typical features of ^3^MLCT emitters, similar to their structural analogs [ReX(CO)_3_(bipy)]. Typically of ^3^MLCT, the emission bands of [ReCl(CO)_3_(terpy-κ^2^N)] remain broad and structureless in both solution and rigid-glass matrix (77 K). The solvent polarity induces bathochromic shift of the emission upon going from chloroform to acetonitrile, and the frozen-state emissions are significantly blue-shifted and show prolonged lifetimes due to the rigidochromic effect [51,52]. Consistent with a larger conjugation of terpy relative to bipy, the solution emission of [ReCl(CO)_3_(terpy-κ^2^N)] appears at slightly longer wavelengths compared to that for [ReCl(CO)_3_(bipy)] (Figure 2). In turn, the frozen-state emission of [ReCl(CO)_3_(terpy-κ^2^N)] is of higher energy relative to that for [ReCl(CO)_3_(bipy)] and better overlaps with the terpy phosphorescence, indicating a larger competition between ^3^MLCT and ^3^IL in the case of the terpy Re(I) complex, as suggested in [39]. A shortening of the emitting triplet-state lifetime (τ = 3.0 ns in CHCl_3_) of [ReCl(CO)_3_(terpy-κ^2^N)] relative to [ReCl(CO)_3_(bipy)] (τ = 51.0 ns in CHCl_3_) was rationalized by the presence of a dangling (non-coordinated) pyridine ring in [ReCl(CO)_3_(terpy-κ^2^N)], resulting in greater complex flexibility [50].

A more comprehensive understanding of excited-state processes in [ReX(CO)_3_(terpy-κ^2^N)] was achieved through femtosecond transient absorption (fs-TA) spectroscopy [48,50]. By analogy to [ReX(CO)_3_(bipy)] [53,54], the TA spectra of [ReX(CO)_3_(terpy-κ^2^N)] were characterized by two positive signals attributed to excited-state absorptions (ESA): a sharp band below 400 nm assigned to the absorption of the bipy/terpy anion radical and a broad absorption in the visible region corresponding to X/L^•−^→Re (Ligand-to-Metal-Charge-Transfer, LMCT) transitions. Based on global lifetime analysis, it was revealed that the optically populated ^1^MLCT state of [ReCl(CO)_3_(terpy-κ^2^N)] underwent femtosecond intersystem crossing (ISC), populating an interligand-localized excited state (^3^IL) and vibrationally hot ^3^MLCT excited states. The former one is converted into ^3^MLCT on a picosecond timescale, and the relaxed ^3^MLCT state decays via minor radiative and major non-radiative pathways to the ground state [50].

**Figure 3:**
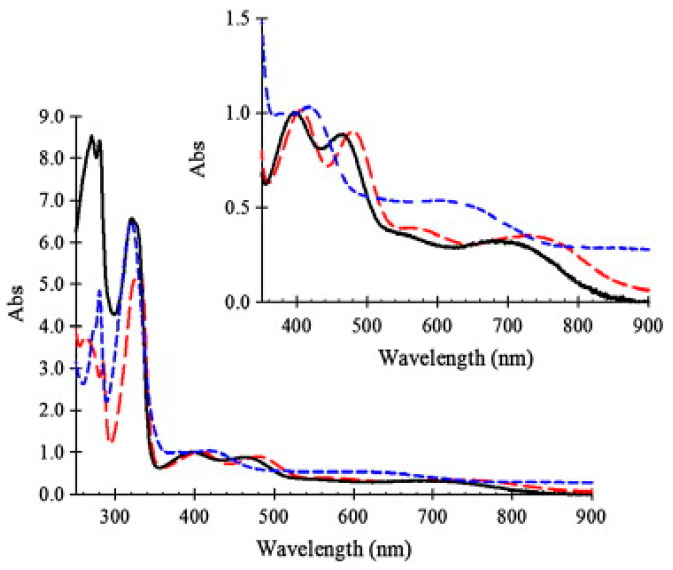
Absorption spectra of [ReCl(CO)_2_(terpy-κ^3^N)] in MeCN (black solid line), CH_2_Cl_2_ (red, long dash) and H_2_O (blue, short dash). Reprinted with permission from Ref. [55]. Copyright 2012 Elsevier.

As reported in [36,47,55], the conversion from the terpy-κ^2^N to terpy-κ^3^N coordination mode results in dramatic changes in the absorption profile, attributed to the significant increase in conjugation due to the planarization of the terpy ligand, and the destabilization of the HOMO level of [ReX(CO)_2_(terpy-κ^3^N)] in relation to that of [ReX(CO)_3_(terpy-κ^2^N)], owing to the replacement of a strongly π-accepting CO group by weakly π-accepting pyridine of the terpy ligand. Regarding the LUMO level, almost no energy changes are observed after the conversion from κ^2^N to the κ^3^N coordination mode. Consistent with the reduced HOMO–LUMO energy gap, the longest wavelength absorption band of [ReX(CO)_2_(terpy-κ^3^N)] exhibits a significant bathochromic shift relative to that of [ReX(CO)_3_(terpy-κ^2^N)]. The complexes [ReX(CO)_2_(terpy-κ^3^N)] are rare examples of dyes that display panchromatic absorption, occurring across the entire visible range of 400–800 nm (Table 3 and Figure 3). Based on the solvent sensitivity of the absorption bands, comparative analysis with the absorption features of the free ligand and theoretical calculations, it was found that intense absorptions in the range of 200–300 nm are best represented by IL transitions, while three broad bands in the visible part of the spectrum of [ReX(CO)_2_(terpy-κ^3^N)] correspond to MLCT transitions.

Conversely to [ReX(CO)_3_(terpy-κ^2^N)], the emission spectrum of [ReX(CO)_2_(terpy-κ^3^N)] was only recorded at 77 K in a 4:1 ethanol–methanol glass. Typically of Re-based ^3^MLCT emitters, the frozen emission band was structureless [36]. In solution at RT, [ReX(CO)_2_(terpy-κ^3^N)] appeared to be non-emissive up to 800 nm [36,47]. According to density functional theory (DFT) and time-dependent density functional theory (TD-DFT) calculations, the emission of Re(I) complexes with *meridionally*-coordinated terpy is predicted at wavelengths longer than 900 nm [47], but experimentally has not been evidenced so far.

## 4. Rhenium(I) Tricabonyl Complexes with 4′-Subsituted 2,2′:6′,2″-Terpyridine Derivatives: Substituent Effects

Structural variations in ligands induce alternations in electron density distribution and spatial configuration of the resulting metal complexes, providing an opportunity to optimize their ground- and excited-state properties, and thus their functional properties. Thanks to a highly efficient Krönhke methodology [56,57], 2,2′:6′,2″-terpyridines substituted at the 4-position of the central pyridine ring (R-terpys) are among the most extensively utilized organic building blocks in coordination chemistry [58]. Since 2013, complexes [ReX(CO)_3_(R-terpy-κ^2^N)] have also been the subject of extensive photophysical characterization.

**Scheme 2:**
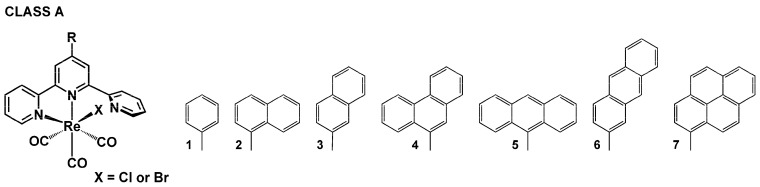
Molecular structures of [ReX(CO)_3_(R-terpy-κ^2^N)] complexes discussed in Section 4.1 (Class A).

**Scheme 3:**
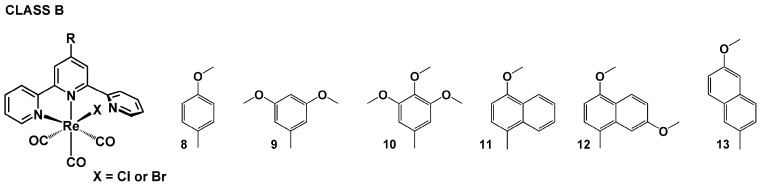
Molecular structures of [ReX(CO)_3_(R-terpy-κ^2^N)] complexes discussed in Section 4.2 (Class B).

**Scheme 4:**
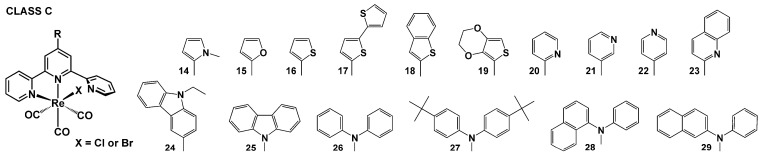
Molecular structures of [ReX(CO)_3_(R-terpy-κ^2^N)] complexes discussed in Section 4.3 (Class C).

**Scheme 5:**
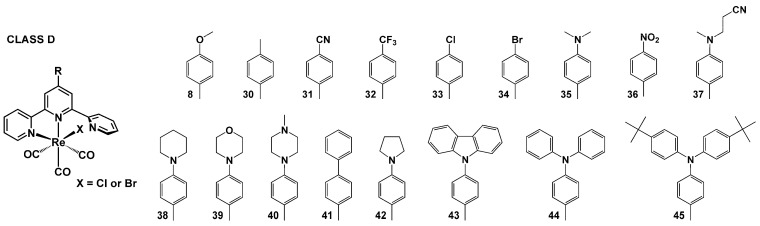
Molecular structures of [ReX(CO)_3_(R-terpy-κ^2^N)] complexes discussed in Section 4.4 (Class D).

Within this review, the impact of 45 chemical motifs introduced into the terpy framework at the 4′-position is analyzed. For a better understanding of the substituent role in controlling the photophysical properties of [ReX(CO)_3_(R-terpy-κ^2^N)], four different classes (Scheme 2, Scheme 3, Scheme 4 and Scheme 5) have been specified, namely (i) [ReX(CO)_3_(R-terpy-κ^2^N)] bearing 2,2′:6′,2″-terpyridines substituted with phenyl and more π-conjugated aryl groups (class A), (ii) [ReX(CO)_3_(R-terpy-κ^2^N)] possessing methoxy-decorated phenyl and naphthyl groups (class B), (iii) [ReX(CO)_3_(R-terpy-κ^2^N)] with heterocyclic or strong electron-releasing groups directly attached to the terpy core at the 4′-position (class C), and (iv) [ReCl(CO)_3_(R-C_6_H_4_-terpy-κ^2^N)] with remote substituents attached via a phenylene bridge to the central pyridine ring of terpy (class D). The photophysics of the above-mentioned systems is discussed in the next Section 4.1, Section 4.2, Section 4.3 and Section 4.4.

### 4.1. Phenyl and More π-Conjugated Hydrocarbon Groups

The relationships between the π-conjugation and linking mode of the aryl hydrocarbon groups attached to the terpy core and the photophysics of resulting complexes [ReCl(CO)_3_(R-terpy-κ^2^N)] were extensively investigated by our research group for the series of chloride complexes **1**–**7** [59,60,61,62].

**Figure 4:**
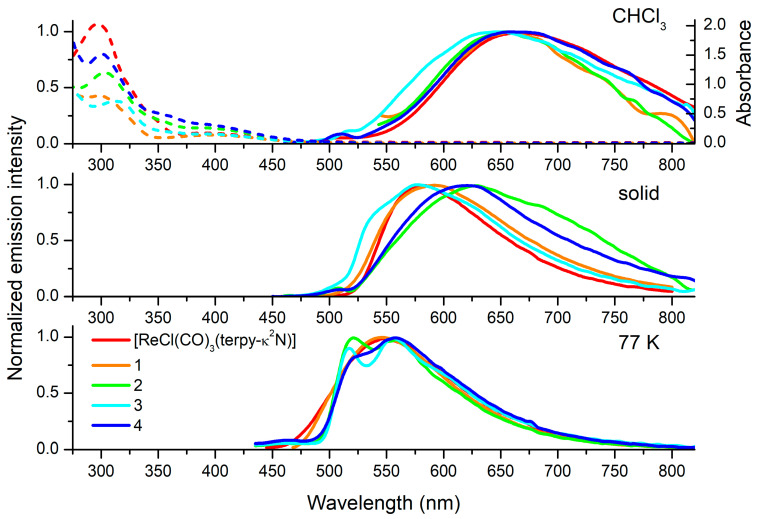
The absorption (dashed lines) and emission (soild lines) properties of [ReCl(CO)_3_(R-terpy-κ^2^N)] with phenyl, naphtyl, and phenanthrenyl pendant groups in relation to the model chromophore [ReCl(CO)_3_(terpy-κ^2^N)]. The spectra are readapted from our previous works [46,50,59,60,61].

Based on UV-Vis absorption spectra, emission wavelengths, and lifetimes, we demonstrated that the complexes [ReCl(CO)_3_(R-terpy-κ^2^N)] with phenyl, naphtyl, and phenanthrenyl pendant groups preserve MLCT character, and the aryl-localized triplet excited state is not accessed upon photoexcitation in these systems. Relative to the model chromophore [ReCl(CO)_3_(terpy-κ^2^N)], the changes in the absorption and solution RT emission maxima are rather negligible; likewise, the lifetimes of **1^Cl^**–**4^Cl^** fall in the nanosecond range. The presence of the π-conjugated naphtyl and phenanthrenyl substituents is generally manifested in a slight intensity increase of the visible light absorption and the appearance of vibrational progression in the frozen emission band (Figure 4 and Table S5). Consistent with the predominant ^3^MLCT character of the lowest triplet state of **1^Cl^**–**4^Cl^** and its destabilization due to an increase in medium rigidity upon cooling [51,52], the frozen-state emission of **1^Cl^**–**4^Cl^** appears in a noticeably higher energy region and shows a significantly prolonged luminescence lifetime in relation to the RT emission in solution.

Further evidence for the formation of the ^3^MLCT state in these systems was provided using fs-TA spectroscopy, carried out for the representative complexes [ReX(CO)_3_(R-terpy-κ^2^N)] with phenyl (**1^Cl^**, **1^Br^**) and 1-naphtyl substituent (**2^Cl^**) [48,59,61], as well as time-resolved infrared spectroscopy performed for **1^Cl^** [63]. All these studies confirmed the formation of the ^3^MLCT state in [ReX(CO)_3_(R-terpy-κ^2^N)] on a picosecond time scale.

The experimental findings were fully supported by DFT calculations, which showed that the attachment of phenyl (**1^Cl^**, **1^Br^**), 1-naphtyl (**2^Cl^**), 2-naphtyl (**3^Cl^**), and 2-triphenylenyl (**4^Cl^**) induced only subtle changes in the HOMO and LUMO energies and character of **1**–**4** relative to [ReX(CO)_3_(terpy-κ^2^N)]. Similarly to the model chromophores [ReX(CO)_3_(terpy-κ^2^N)], the highest occupied molecular orbital (HOMO) of **1**–**4** is principally localized on the {Re(CO)_3_X} unit, and the lowest unoccupied molecular orbital (LUMO) is contributed by the π-antibonding orbitals of the coordinated rings of the terpy core [47,62,64,65]. The HOMO–LUMO energy gaps of **1^Cl^**–**4^Cl^**, varying from 3.79 eV to 3.82 eV, correlate well with the value of 3.89 eV calculated for [ReCl(CO)_3_(terpy-κ^2^N)] [53,59,60]. Some variations in photophysical properties between bromide- and chloride-substituted complexes **1^Cl^** and **1^Br^** (Table S5) were attributed to the halide contribution to the excited state [47].

Distinctly from the Re(I) complexes with phenyl, naphtyl, and phenanthrenyl pendant groups, the complexes [ReCl(CO)_3_(R-terpy-κ^2^N)] with 9-anthryl (**5^Cl^**), 2-anthryl (**6^Cl^**), and 1-pyrenyl (**7^Cl^**) groups are non-emissive in the solid state, and their solution photophysical properties are strongly affected by the aryl substituent [61,62]. As supported by DFT calculations, the HOMO of these systems is localized on the aryl substituent and is effectively destabilized (~0.4 eV) compared to the model chromophore. The LUMO largely resides on the terpy core, and its energy is hardly perturbed by the anthryl and pyrenyl substituents in relation to that for [ReCl(CO)_3_(terpy-κ^2^N)]. A slight stabilization of the LUMO can be noticed upon the replacement of 9-anthryl by 2-anthryl, which was rationalized by a stronger coupling between the 2-anthryl group and the terpy unit. Consistent with the reduced HOMO–LUMO gaps, the lowest energy absorptions of **5^Cl^**–**7^Cl^** have a noticeable red-shift relative to **1^Cl^**. For **6^Cl^** and **7^Cl^**, a bathochromic shift of the longest wavelength absorption is accompanied by a significant increase in its visible absorptivity due to the overlapping of ^1^MLCT and ^1^ILCT/^1^IL transitions. A large dihedral angle between the appended 9-anthryl and central pyridine ring of terpy results in the separation of ^1^MLCT and ^1^IL states in **5^Cl^**.

The complex [ReCl(CO)_3_(R-terpy-κ^2^N)] with the pendant pyrenyl group (**7^Cl^**) was demonstrated to exhibit “ping-pong” energy transfer. Its excitation leads to a predominant population of the ^1^ILCT state, which undergoes energy transfer to the ^1^MLCT* state via the Förster resonance energy transfer (FRET) mechanism. In the next step, the ^1^MLCT is converted to the ^3^MLCT* by femtosecond intersystem crossing (ISC), and the formed ^3^MLCT is further relaxed to the lower energy triplet excited state localized on the pyrenyl-terpy ligand. Also, the time-resolved emission spectra at 77 K and ns-TA spectroscopy confirmed that the T_1_ state of **7^Cl^** is localized on the pyrenyl moiety. Due to the energetic proximity of the ^3^MLCT and ^3^IL/^3^ILCT excited states, and the establishment of the triplet-state equilibrium between them, the complex **7^Cl^** shows a prolonged triplet excited-state lifetime at RT (4.4 μs) [61]. The pyrene chromophore in **7^Cl^** acts as an energy reservoir for ^3^MLCT [65,66,67,68,69,70].

Also, complexes **5^Cl^** and **6^Cl^** were found to exhibit a substantial enhancement of RT lifetimes in DMSO solution as a result of accessing the low-lying ^3^IL state of the anthracene chromophore. Using steady-state and time-resolved optical techniques, our group demonstrated the impact of the different relative orientations of anthracene and {ReCl(CO)_3_(terpy-κ^2^N)} chromophores on the photophysical behavior of the resulting complexes, **5^Cl^** and **6^Cl^**. A more planar geometry of 2-anthryl-terpy, and thus stronger overlapping orbitals of 2-anthryl and terpy moieties, was evidenced to facilitate the population of the anthracene triplet excited state, leading to the prolongation of its lifetime. The phosphorescence lifetimes of **5^Cl^** and **6^Cl^** were determined as 14.28 *μ*s for **5^Cl^** and 22.71 *μ*s for **6^Cl^**. It should be noted that transition metal complexes with extended emission lifetimes are strongly desirable for applications involving intermolecular photoinduced energy triplet state transfer, such as photodynamic therapy (PDT), time-resolved bioimaging, or triplet–triplet annihilation up-conversion (TTA UC) [19,69,71,72,73,74,75,76,77]. The suitability of **5^Cl^** and **6^Cl^** to transfer the excited triplet state energy to molecular oxygen was confirmed in our studies [62], demonstrating a slightly enhanced singlet oxygen sensitizing ability of **6^Cl^** (Φ_ΔO_2__ = 0.45) in relation to **5^Cl^** (Φ_ΔO_2__ = 0.42). Importantly, the complexes [ReCl(CO)_3_(R-terpy-κ^2^N)] with 9-anthryl (**5^Cl^**) and 2-anthryl (**6^Cl^**) were demonstrated to be rare examples that show both ^3^MLCT and ^3^anthracene emission. Consequently, their DMSO solution and electroluminescence spectra cover a broad range from 500 nm to the near-infrared region of 700–900 nm. The addition of a component with an emission from 400 to 500 nm might yield a diode, which emits white light [62].

### 4.2. Methoxy-Decorated Phenyl and Naphthyl Groups

The photophysical properties of [ReX(CO)_3_(R-terpy-κ^2^N)] with methoxy-decorated phenyl and naphthyl groups (class B) were the subject of the research reported in [23,78,79,80].

The attachment of one or more methoxy groups was found to induce only subtle variations in the solution emission properties of **8^Cl^**–**13^Cl^** relative to those for corresponding model chromophores **1^Cl^**–**3^Cl^**, implying that the emitting state is of ^3^MLCT nature in all these systems. As shown in Table S6, the wavelength maxima of the broad and structureless emission bands of **8^Cl^**–**13^Cl^** in acetonitrile and chloroform fall in a narrow range of 645–675 nm, lifetimes are in the nanosecond domain, and emission quantum yields are below 1.5%, similarly to features of model chromophores **1^Cl^**–**3^Cl^**.

Conversely, the solid-state emission of **8^Cl^**–**13^Cl^** was evidenced to be strongly affected by the number of methoxy groups and their substitution pattern. The bathochromic shift of the solid-state emission maximum follows the order 4-methoxy-1-naphthyl (**11^Cl^**, λ_em_ = 574 nm) < 3,4,5-trimethoxy-1-phenyl (**10^Cl^**, λ_em_ = 580 nm) < 4-methoxy-1-phenyl (**8^Cl^**, λ_em_ = 584 nm) < 3,5-dimethoxy-1-phenyl (**9^Cl^**, λ_em_ = 600 nm) < 4,7-dimethoxy-1-naphthyl (**12^Cl^**, λ_em_ = 633 nm) ~ 6-methoxy-2-naphthyl (**13^Cl^**, λ_em_ = 631 nm), indicating that the methoxy group at the para position of the phenyl/naphtyl ring attached to the central pyridine ring of terpy tends to induce a larger hypsochromic shift of the emission. These findings were rationalized by the fact that the electron-rich methoxy group at the para position is known to add electron density into π-acceptor moiety, leading to the destabilization of the ^3^MLCT excited state in resulting transition metal complexes. In contrast, meta-positioned –OCH_3_ groups are expected to withdraw electron density [81]. Consistent with the rigidochromic effect, the solid emission spectra of **8^Cl^**–**13^Cl^** were blue-shifted compared to those in solution (Table S6).

Noticeable differences were also observed in the solid-state emission lifetimes of **8^Cl^**–**13^Cl^**, which varied from nano- to microseconds. Relative to the model chromophores **1^Cl^**–**3^Cl^**, a substantial prolongation of the solid-state emission lifetime was evidenced for complexes **8^Cl^**, **10^Cl^**, **11^Cl^**_,_ and **13^Cl^**. The triplet excited-state lifetimes were extended to 586 ns for **8^Cl^**, 446 ns for **10^Cl^**, 8.62 μs for **11^Cl^**, and 49.62 μs for **13^Cl^**, compared to 52 ns for **1^Cl^**, 162 ns for **2^Cl^**, and 102 ns for **3^Cl^**. Except for **12^Cl^**, the solid-state emission lifetimes of the Re(I) complexes belonging to class B underwent extension with an increase in π-conjugation of the pendant aryl group. Lowering the temperature down to 77 K resulted in a further increase in their excited-state lifetimes and a blue-shift of the emission maxima. As supported theoretically [78,79,82], methoxy-decoration of naphthyl groups resulted in an increased contribution of the organic ligand in the ground and excited states of resulting [ReX(CO)_3_(R-terpy-κ^2^N)], which may explain their prolonged solid-state emission lifetimes. To obtain triplet emitters with long excited states, both radiative and non-radiative decay rate constants must be small, meaning that the triplet excited state has a predominant ligand character [83].

Regarding the solid-state emission quantum yield, the complex [ReCl(CO)_3_(R-terpy-κ^2^N)] with 4-trimethoxy-1-phenyl substituent (**8^Cl^**) was found to be outstanding. Its emission quantum yield of ~30% is superior to the values recorded for chloride systems **1**–**3** and **9**–**13** (Tables S5 and S6).

Having good thermal properties and suitable energy levels, IP, and EA, the compounds **8^Cl^**–**13^Cl^** were employed as active layers in light-emitting diodes ITO/PEDOT:PSS/compound/Al and ITO/PEDOT:PSS/PVK:PBD:compound/Al, fabricated in the laboratory. Most of the obtained devices with the Re(I) complexes exhibited orange or red emission under external voltage [78,79,82]. The authors of [82] demonstrated the additional possibility of electroluminescence enhancement by incorporating silver nanowires (AgNWs) into the PEDOT:PSS layer.

### 4.3. Heterocyclic or Strong Electron-Releasing Groups Directly Attached to the Terpy Core at 4′-Position

Among the compounds **14^Cl^**–**24^Cl^ [37,47,50,84,85]**, outstanding photophysical behavior was revealed for the chloride Re(I) complex with the strong electron-donating bithiophene substituent (**17^Cl^**).

**Figure 5:**
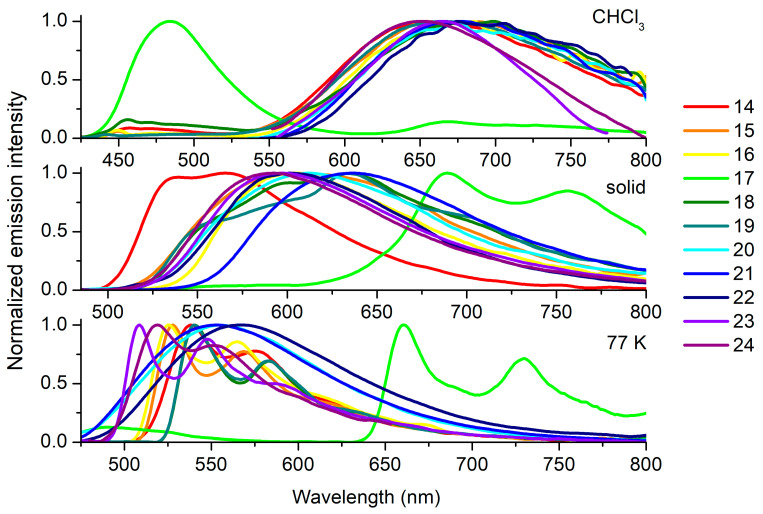
The normalized emission spectra of **14^Cl^**–**24^Cl^** in CHCl_3_, solid state and frozen matrices at 77 K. The spectra are readapted from our previous works [50,84,85].

Its emission profile was found to be markedly distinct in comparison to other compounds of this class. As depicted in Figure 5, the emission spectrum of **17^Cl^** in CHCl_3_ is primarily dominated by the ^1^ILCT fluorescence band. The lower energy phosphorescence band of **17^Cl^** exhibits a weak vibronic structure, and it is nearly overlapping with the solid-state and frozen ligand-centered emission. The triplet-emitting state of **17^Cl^** is of ^3^ILCT nature, likely originating from optically populated ^1^ILCT. Both fluorescence and phosphorescence are discernible in the solution and frozen matrix emission spectra. Compared to **14^Cl^**–**16^Cl^** and **18^Cl^**–**24^Cl^**, the absorption and phosphorescence bands of **17^Cl^** appear at significantly lower energies (Figure 5 and Table S7). In the solid state and EtOH–MeOH (4:1) glass matrix, the triplet excited-state lifetimes of **17^Cl^** are extended to 24.4 μs and 178 μs, respectively.

Using static and time-resolved emission spectroscopy, ultrafast transient absorption measurements, and DFT/TD-DFT calculations [37,47,50,84,85], the emitting excited states of **14^Cl^**–**16^Cl^** and **18^Cl^**–**24^Cl^** were demonstrated to have a predominant ^3^MLCT character. A significantly noticeable effect on the position of the emission band in solution was observed for complexes bearing electron-donating groups, namely **14^Cl^** in both MeCN and CHCl_3_, **18^Cl^** in MeCN, and **19^Cl^** in MeCN (Table S7), compared to the model chromophore [ReCl(CO)_3_(terpy-κ^2^N)]. In the research work [85], the variations in the emission position of **20^Cl^**–**22^Cl^** were correlated with Hammett σ parameters, demonstrating a decrease in the emission energy in accordance with the increased electron-withdrawing properties of the pendant *n*-pyridyl groups attached to terpy. For all complexes **14^Cl^**–**16^Cl^** and **18^Cl^**–**24^Cl^**, the emission lifetimes in solution are in the nanosecond domain, and quantum yields are below 1%. Furthermore, typically of the emission from a ^3^MLCT state, the frozen and solid-state emission of **14^Cl^**–**16^Cl^** and **18^Cl^**–**24^Cl^** occurs in a higher energy region, showing prolonged lifetimes with reference to the solution (Table S7).

Analogously to the compounds of class B, the solid-state photo-characteristics of **14^Cl^**–**24^Cl^** were noticeably affected by substituents at the 4′-position of terpy. The solid emission wavelengths varied with the terpy substituents from 543 nm for **14^Cl^** to 636 nm for **21^Cl^**, confirming the key role of the donor-acceptor abilities of the pendant substituent. A substantial prolongation of the solid-state emission lifetimes was confirmed for **14^Cl^** (11.8 μs), **18^Cl^** (5.2 μs), and **19^Cl^** (17.0 μs). As supported theoretically [37], the appended N-methyl-pyrrole, benzothiophene, and ethylenedioxythiophene electron-donating groups induce an enhanced contribution of the organic ligand in the ground and excited states of the resulting [ReX(CO)_3_(R-terpy-κ^2^N)]. Enhanced emission efficiency in the solid state (above 10%) was found for compounds **19^Cl^** and **24^Cl^**.

The capability of **14^Cl^**–**24^Cl^** for the emission of light under voltage was examined in [50,84,85], and the fabricated diodes ITO/PEDOT:PSS/PVK:PBD:complex/Al were found to emit light under the applied voltage, with maximum electroluminescence falling in the light range from yellow to red.

The photophysical properties of **25^Cl^**–**29^Cl^** were the subject of experimental studies reported in [49]. It was evidenced that the hole-transporting carbazole and diphenylamine moieties, directly attached to the central pyridine of the terpy core, favor the energy transfer process from the substituent to the terpy core, leading to an enhancement of the visible absorptivity and luminescence performance of the resulting [ReX(CO)_3_(R-terpy-κ^2^N)] complexes. The emission performances of **26^Cl^**–**28^Cl^** largely exceeded that of the model chromophore [ReCl(CO)_3_(terpy-κ^2^N)] (Table S7). The phosphorescence maximum of **26^Cl^**–**28^Cl^** appears in the range of 578–601 nm in CH_2_Cl_2_ solution and 533–611 nm in the solid state. In solution, the emission lifetime decays are in the microsecond domain, and emission quantum yields vary from 0.3% to 1.3%.

The photophysical properties of **25^Cl^**–**29^Cl^** were also investigated theoretically, and computed ground- and excited-state properties were analyzed in terms of the potential utility of these systems for OLED technology [86]. The visible absorption of **25^Cl^**–**29^Cl^** was assigned to electronic transitions of mixed ^1^MLCT/^1^LLCT/^1^ILCT character, while the phosphorescence was associated with ^3^MLCT/^3^LLCT/^3^ILCT triplet states. As supported by the reorganization energy calculations, the carbazole and diphenylamine moieties noticeably improve the electron transport performance of the resulting Re(I) complexes, and **25^Cl^**–**29^Cl^** can be regarded as suitable candidates for OLED materials.

### 4.4. [ReX(CO)_3_(R-C_6_H_4_-terpy-κ^2^N)] with Remote Substituents Attached via a Phenylene Bridge to the Central Pyridine Ring of Terpy

The remote substituent impact in [ReX(CO)_3_(R-C_6_H_4_-terpy-κ^2^N)] was explored by the Fernández-Terán [63,64], Hanan [47], and Machura [46,59,60,78,82,87,88] research groups (Table S8). The authors of [63] conducted a comprehensive investigation of a series of complexes **1^Cl^**, **8^Cl^**, **31^Cl^**, **32^Cl^**, **34^Cl^**, **35^Cl^**, and provided definitive experimental evidence for a change in the excited-state character from ^3^MLCT (**1^Cl^**, **8^Cl^**, **31^Cl^**, **32^Cl^** and **34^Cl^**) to ^3^ILCT in [ReX(CO)_3_(R-C_6_H_5_-terpy-κ^2^N)] with the strongest electron-releasing substituent, –NMe_2_ (**35^Cl^**).

The optical properties of **1^Cl^**, **8^Cl^**, **31^Cl^**, **32^Cl^**, **34^Cl^** were found to be systematically varied with electron-donating substituent abilities, as demonstrated by a hypsochromic shift of the ^1^MLCT absorption and ^3^MLCT emission bands with the increased electron-donating character of the substituent, linear correlations between the ^3^MLCT lifetimes and Hammett σ_p_ substituent constants, and linear correlations of ΔG_S-T_ values obtained from a linear fit of the high-energy side of the low-temperature emission spectra with the Hammett σ_p_ substituent constants. At RT, all these systems exhibit broad and unstructured emission, with lifetimes varying from 0.58 to 2.3 ns between the CN- and OMe-substituted complexes. Typically of ^3^MLCT emitters, their photoluminescence spectra show significant hypsochromic shifts in solid states and at cryogenic temperatures (77 K).

Markedly different absorption and emission properties were demonstrated for **35^Cl^**. Most importantly, the change in the singlet and triplet excited-state character from MLCT to ILCT was manifested in the appearance of a very strong visible absorption band, red-shifted by ~100 nm relative to **1^Cl^**, dramatic enhancement of the excited-state lifetime in solution (380 ns in DMF), and a bathochromic shift of the emission upon cooling, accompanied by the appearance of a vibronic structure. Clear differences between ^3^MLCT and ^3^ILCT Re(I) emitters belonging to this group were also evidenced in photocatalytic hydrogen evolution experiments, performed for **8^Cl^** and **35^Cl^** as representative photosensitizers [63]. Contrary to **8^Cl^**, which induced stable hydrogen evolution with a turnover number (TON) for the photosensitizer of 580 ± 40, the use of **35^Cl^** with 10-fold-smaller photosensitizer concentrations resulted in very fast hydrogen evolution, with TONs of over 2100. The complex **35^Cl^** was also demonstrated to possess superior capability for ^1^O_2_ generation and release of CO under ultrasound irradiation. Its excellent sonocytotoxicities towards both normoxic and hypoxic cancer cells were confirmed in *in vitro* and *in vivo* experiments. Notably, the complex **35^Cl^** had significant advantages in sonocytotoxicity relative to its analog with electron-withdrawing **36^Cl^**, characterized by considerably lower luminescence intensity, shorter lifetime, and smaller ^1^O_2_ quantum yield [89].

In the paper [64], Fernández-Terán and co-workers presented a theoretical and experimental comparative analysis of the one-photon and two-photon absorption properties of **8^Cl^** and **35^Cl^**. Their studies revealed that the nonlinear behavior of [ReX(CO)_3_(R-C_6_H_4_-terpy-κ^2^N)] is predominately governed by the conjugation size of the aromatic system, while the increased charge-transfer character of the excited states plays a minor role in the two-photon absorption behavior, contrary to the one-photon properties of these systems.

The effect of the conjugation degree and electron-donating ability of remote groups in controlling ground- and excited-state properties of [ReX(CO)_3_(R-C_6_H_4_-terpy-κ^2^N)] was demonstrated for compounds **42^Cl^** and **43^Cl^** in [59,60]. In contrast to five-membered pyrrolidine, the N-carbazolyl is also able to accept electron density due to the presence of two benzene rings fused on either side of the heterocyclic amine ring. Based on transient absorption studies in nano- and femtosecond domains, it was demonstrated that the attachment of 9-carbazole via the phenylene bridge to the terpy core did not lead to a switch in the excited-state character of [ReX(CO)_3_(R-C_6_H_4_-terpy-κ^2^N)] from ^3^MLCT to ^3^ILCT. The emission of **43^Cl^** was largely superimposed with the band of **1^Cl^** in solution and rigid-glass matrix at 77 K. In turn, the introduction of the electron-donating pyrrolidine group resulted in different emission behavior of **42^Cl^** in acetonitrile and chloroform solutions, showing a red- and blue-shift in relation to the reference chromophore **1^Cl^**, respectively. Upon cooling to 77 K, the complex **42^Cl^** showed a clear bathochromic shift relative to the model chromophore. Similar trends were also observed for **38^Cl^**, **39^Cl^**, **43^Cl^**, and **44^Cl^**, indicating the presence of ^3^MLCT to ^3^ILCT in energy proximity in [ReX(CO)_3_(R-C_6_H_4_-terpy-κ^2^N)] with electron-donating substituents (Table S8).

The authors of [88] conducted comprehensive studies of photoinduced processes in **37^Cl^** and **40^Cl^** depending on the polarity of the environment, confirming the change in the nature of the triplet excited state from ^3^MLCT to ^3^ILCT in polar solvents. A bathochromic shift in the emission position of these systems in polar solvents was accompanied by the enhancement of the excited-state lifetime relative to the unsubstituted chromophore **1^Cl^** (Table S8).

## 5. Substituent Effect in Rhenium(I) Dicarbonyl Complexes with *Meridionally*-Coordinated 4′-Subsituted 2,2′:6′,2″-Terpyridines: The Impact of the Coordination Mode

**Scheme 6:**
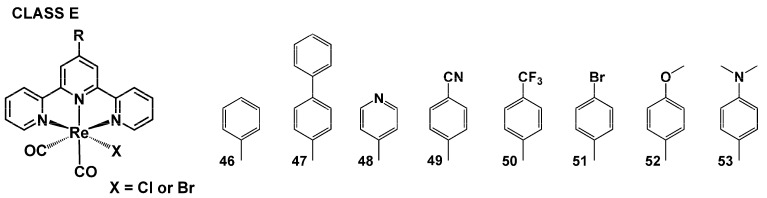
Molecular structures of [ReX(CO)_2_(R-terpy-κ^3^N)] complexes discussed in this section (Class E).

The ground and excited states of [ReX(CO)_2_(R-terpy-κ^3^N)] with 4′-subsituted 2,2′:6′,2″-terpyridines (Scheme 6 and Table S9) were evaluated in [37,47,80]. Importantly, the investigations of the Fernández-Terán [80] and Hanan [47] research groups are complementary to those for [ReX(CO)_2_(R-C_6_H_5_-terpy-κ^2^N)], making it possible to establish the impact of the coordination mode on the photophysical properties of terpy-based Re(I) carbonyl complexes.

By analogy to the model chromophores [ReX(CO)_2_(terpy-κ^3^N)] (see Section 3), all Re(I) complexes with *meridionally*-coordinated R-terpys absorb in the entire visible region, exhibiting three strong bands at approximately 720 nm, 480 nm, and 410 nm, assigned experimentally and theoretically to ^1^MLCT transitions. For complexes **46^Br^**, **47^Br^**, and **48^Br^**, only a slight red-shift is observed relative to [ReBr(CO)_2_(terpy-κ^3^N)], attributed to the extended conjugation following the introduction of the aromatic substituent [47]. As reported by the Fernández-Terán group [80], the complexes **46^Cl^**, **49^Cl^**–**53^Cl^** exhibit small hypsochromic shifts in the visible absorption maxima with an increased electron-donating character of the substituent, accompanied by a noticeable increase in the extinction coefficients for [ReX(CO)_3_(R-C_6_H_4_-terpy-κ^2^N)] with the strongest electron-releasing substituent –NMe_2_ (**53^Cl^**). In contrast to **35^Cl^**, however, the visible absorptions of **53^Cl^** are not contributed by ILCT transitions.

Conversely to [ReX(CO)_3_(R-C_6_H_4_-terpy-κ^2^N)], all halide Re(I) complexes with *meridionally*-coordinated 4′-substituted-terpyridines are non-emissive in solution at RT, which was attributed to fast non-radiative deactivation, in agreement with significantly red-shifted absorption.

Based on the time-resolved infrared (TRIR) spectra, Fernández-Terán and co-workers [80] evidenced that the photophysical properties of **46^Cl^**, **49^Cl^**–**53^Cl^** are governed by the ^3^MLCT excited state, independent of the substituent on the terpy core. The ^3^MLCT excited state evolves from the optically populated ^1^MLCT state via intersystem crossing, and complexes **46^Cl^**, **49^Cl^**–**53^Cl^** show linear correlations between the ^3^MLCT lifetimes and Hammett σ_p_ substituent constants. The studies demonstrated clearly that the presence of the electron-rich {Re(CO)_2_}^+^ moiety in [ReX(CO)_2_(R-terpy-κ^3^N)] systems hinders access to ILCT excited states.

## 6. The Effect of the Ancillary Ligand

**Scheme 7:**
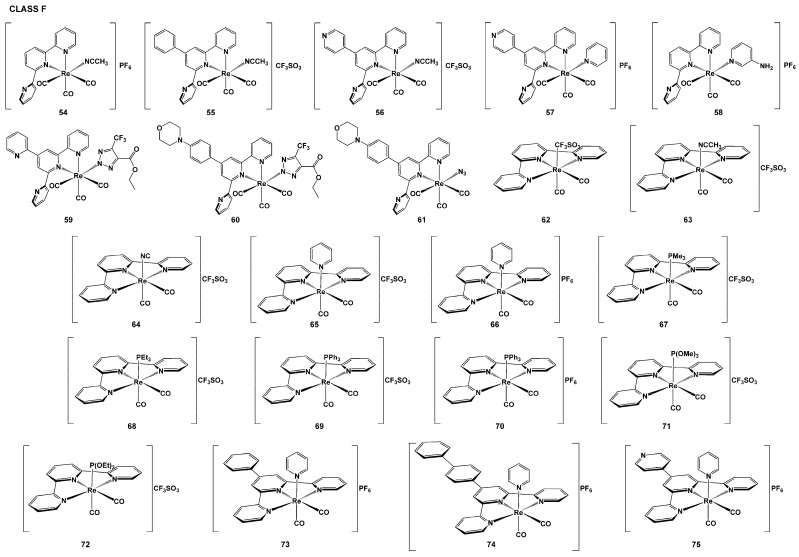
Molecular structures of [Re(X/L)(CO)_3_(R-terpy-κ^2^N)]^0/+^ and [Re(X/L)(CO)_2_(R-terpy-κ^3^N)]^0/+^ complexes discussed in Section 6. (Class F). X is a mono-negative anion different from Cl and Br, and L stands for a neutral ligand.

The impact of varying the ancillary ligand nature on the photophysical properties of Re(I) carbonyl complexes with R-terpy remains rather unexplored. The findings in this field were reported in [36,47,55,90,91,92,93]. The complexes [Re(X/L)(CO)_3_(R-terpy-κ^2^N)]^0/+^ and [Re(X/L)(CO)_2_(R-terpy-κ^3^N)]^0/+^ bearing ancillary ligands different from halide ions (Scheme 7, Class F) exhibit typical absorption profiles for [Re(Cl/Br)(CO)_3_(R-terpy-κ^2^N)] and [Re(Cl/Br)(CO)_2_(R-terpy-κ^3^N)], respectively.

According to TDDFT calculations and experimental findings, all visible absorptions of [Re(X/L)(CO)_2_(R-terpy-κ^3^N)]^0/+^ are purely MLCT in nature, while the longest wavelength absorption band of [Re(X/L)(CO)_3_(R-terpy-κ^2^N)]^0/+^ generally has mixed MLCT/LLCT/IL character, but with a predominant contribution of MLCT transitions [36,47,91]. As shown in Table S10, the replacement of halide ions by neutral N- and P-donor ligands generally leads to blue-shift of the lowest energy absorption, and much more profound shifts are noticeable for Re(I) complexes with *meridionally*-coordinated terpys. For example, the lowest energy band of **66** and **70** appears at ~40 and ~100 nm shorter wavelengths relative to [ReBr(CO)_2_(terpy-κ^3^N)], respectively [47].

The emission spectra of [Re(X/L)(CO)_3_(R-terpy-κ^2^N)]^0/+^ in solution at RT were recorded only for 54 and 57 [39,91]. Consistent with a blue-shift observed in the absorption spectra, their emission appears in a higher energy region relative to [ReCl(CO)_3_(terpy-κ^2^N)] and [ReCl(CO)_2_(py-terpy-κ^3^N)] (22), respectively (Table S10). The complexes 53 and 56 emit below 600 nm, showing a broad and structureless band, typically of the emission originating from the lowest energy ^3^MLCT excited state.

Most importantly, the complexes **66**, **70**, **73**, **74**, and **75** emit in a NIR range, with the emission maximum at 870 nm, 800 nm, 876 nm, 865 nm, and 950 nm, respectively. The emission quantum yields of **66**, **70**, **73**, **74**, and **75** fall in the range of 0.76–0.02, while the lifetime was able to record only for **70** (10.2 ns). The emission of these systems occurs from the ^3^MLCT excited state [47,91]. Since cells and tissues show negligible absorption and autofluorescence in the NIR range, metal complexes emitting above 750 nm are of particular significance in view of their potential in biomedical molecular imaging **[94,95,96,97,98,99,100]**.

## 7. Insight into the Molecular Structures of [Re(X/L)(CO)_3_(R-terpy-κ^2^N)]^0/+^ and [Re(X/L)(CO)_2_(R-terpy-κ^3^N)]^0/+^ from Electrochemistry

**Figure 6:**
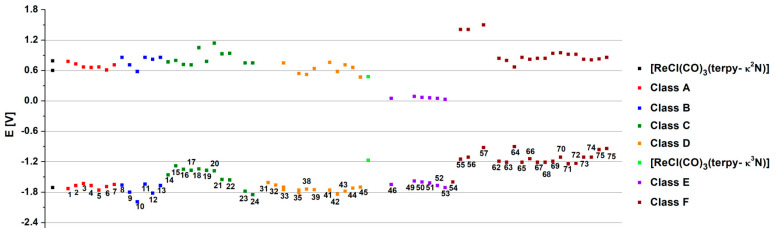
The first oxidation and reduction potentials of [Re(X/L)(CO)_3_(R-terpy-κ^2^N)]^0/+^ and [Re(X/L)(CO)_2_(R-terpy-κ^3^N)]^0/+^ determined by cyclic voltammetry.

The electrochemical properties of [Re(X/L)(CO)_3_(R-terpy-κ^2^N)]^0/+^ and [Re(X/L)(CO)_2_(R-terpy-κ^3^N)]^0/+^ were examined using cyclic voltammetry by several groups [32,36,39,46,47,48,49,50,59,60,61,62,63,78,79,80,82,84,85,87,90,91]. The electrochemical data are summarized in Table S11, and the first oxidation and reduction potentials are presented in Figure 6. For the vast majority of the halide Re(I) carbonyl complexes, the first reduction peak, associated with the terpy-centered reduction, is reversible or quasi-reversible and falls in a potential range varying from −1.65 to −1.85 V versus the ferrocene/ferrocenium redox couple_._ Slightly more positive E_red_ potentials were confirmed for compounds **14^Cl^**–**22^Cl^** bearing heterocyclic substituents, while a more noticeable cathodic shift was revealed for [ReCl(CO)_3_(R-terpy-κ^2^N)] with pendant 3,4,5-trimethoxy-1-phenyl group (**10^Cl^**). The replacement of the halide ion by the N- or P-donor ligand results in anodic shifts of reduction potentials. As evidenced in [47,60,79,80,87], the first reduction potentials of [ReX(CO)_3_(R-terpy-κ^2^N)] and [ReX(CO)_2_(R-terpy-κ^3^N)] become slightly more negative with more electron-donating substituents, while electron-accepting groups make Re(I) carbonyl complexes easier to reduce. Rather small variations in E_red_ potentials are observed between Re(I) complexes bearing the same ligand but differing in the terpy coordination mode. In contrast, the oxidation potentials of the halide systems [ReX(CO)_3_(R-terpy-κ^2^N)] and [ReX(CO)_2_(R-terpy-κ^3^N)] are most affected by the terpy coordination mode. The conversion from terpy-κ^2^N to terpy-κ^3^N results in significant anodic shifts of E_ox_ potentials, as well as modifying the reversibility, making the oxidation processes reversible in [ReX(CO)_2_(R-terpy-κ^3^N)]. Compared to [ReX(CO)_3_(R-terpy-κ^2^N)] and [ReX(CO)_2_(R-terpy-κ^3^N)], the cationic complexes [ReL(CO)_3_(R-terpy-κ^2^N)]^+^ and [ReL(CO)_2_(R-terpy-κ^3^N)]^+^ are noticeably harder to oxidize. The cationic Re(I) tricarbonyl complexes with R-terpy-κ^2^ are the most difficult to oxidize (**55**–**57**). For all complexes [Re(X/L)(CO)_3_(R-terpy-κ^2^N)]^0/+^ and [Re(X/L)(CO)_2_(R-terpy-κ^3^N)]^0/+^, oxidation potentials slightly decrease upon the attachment of pendant electron-donating groups to the terpy framework [36,47,60,63,79,80,87].

**Figure 7:**
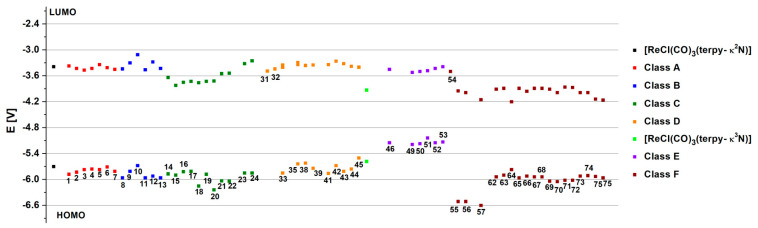
The HOMO and LUMO energy levels of [Re(X/L)(CO)_3_(R-terpy-κ^2^N)]^0/+^ and [Re(X/L)(CO)_2_(R-terpy-κ^3^N)]^0/+^, estimated on the basis of potentials of the oxidation and reduction couples with regard to the energy level of the ferrocene reference: HOMO energy levels estimated from equation IP= −5.1 − E_ox_; LUMO energy levels estimated from equation EA= −5.1 − E_red_.

The HOMO and LUMO energy levels of [Re(X/L)(CO)_3_(R-terpy-κ^2^N)]^0/+^ and [Re(X/L)(CO)_2_(R-terpy-κ^3^N)]^0/+^, estimated on the basis of the potentials of the oxidation and reduction couples with regard to the energy level of the ferrocene reference [101,102], reproduce well the trends observed in their absorption spectra and as predicted by DFT/TDDFT calculations (Section 4 and Section 5). The most reduced HOMO–LUMO gaps, reflected experimentally in a significant red-shift of the lowest energy absorption, is observed for [ReX(CO)_2_(R-terpy-κ^3^N)]. The conversion of the coordination mode from terpy-κ^2^N to terpy-κ^3^N leads to a significant destabilization of the HOMO levels in halide Re(I) carbonyl complexes. To a much smaller extent, the HOMO energy level is destabilized by the appended electron-donating groups in the halide systems [ReX(CO)_3_(R-terpy-κ^2^N)], while the formation of cationic complexes [ReL(CO)_3_(R-terpy-κ^2^N)]^+^ and [ReL(CO)_2_(R-terpy-κ^3^N)]^+^ results in the stabilization of both HOMO and LUMO levels (Figure 7).

## 8. Higher Nuclearity Coordination Systems with {Re(CO)_3_(R-terpy-κ^2^N)} and {Re(CO)_2_(R-terpy-κ^3^N)} Motives

**Scheme 8:**
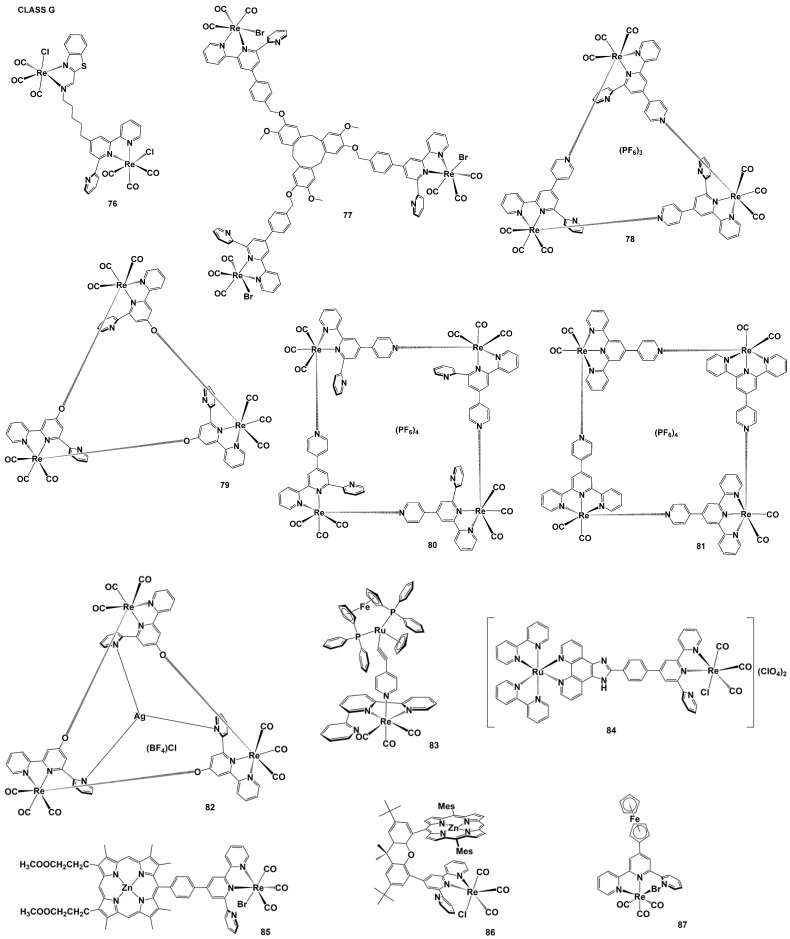
Higher nuclearity coordination compounds including photoactive {Re(CO)_3_(R-terpy-κ^2^N)} and {Re(CO)_2_(R-terpy-κ^3^N)} motives (Class G).

The photoactive {Re(CO)_3_(R-terpy-κ^2^N)} and {Re(CO)_2_(R-terpy-κ^3^N)} units were also utilized to build higher nuclearity coordination compounds [37,48,103,104,105,106,107,108,109]. For the preparation of bi-, tri-, and multicomponent systems with {Re(CO)_3_(R-terpy-κ^2^N)} and {Re(CO)_2_(R-terpy-κ^3^N)} motives (Scheme 8), different synthetic strategies may be employed, namely (i) structural modifications of the terpy framework aiming at the introduction of additional binding sites, (ii) attachment of the terpy core to organic ligands showing higher coordination preferences for other transition metals, and (iii) abstraction of the halide ion from [ReX(CO)_3_(R-terpy-κ^2^N)] and [ReX(CO)_2_(R-terpy-κ^3^N)] by a silver salt followed by coordination of the other component. Noteworthy is that the integration of different metal centers into one system may provide complementary functionalities, as well as result in photoinduced electron or energy transfer processes between metal centers.

As shown in Table S12, the Re-based multicomponent systems exhibit photophysical properties typical of the corresponding mononuclear compounds [ReCl(CO)_3_(R-terpy-κ^2^N)] (**76**–**80**) and [ReCl(CO)_2_(R-terpy-κ^3^N)] (**81**). The complex **81** with *meridionally*-coordinated terpy absorbs across the entire UV-Vis range, up to 800 nm, and emits in the NIR region, with a maximum of 980 nm [91]. The homonuclear systems with R-terpy-κ^2^N (**76**–**80**) absorb energy in a much narrower range of wavelengths, and their emission maxima fall in the range of 460–650. By analogy to the mononuclear chromophores, the absorptions of **76**–**81** occurring above 350 nm are predominantly of a MLCT nature, while higher energy bands in UV-Vis spectra correspond to ligand-centered transitions [91,103,108,109]. The emission of **76**, **78**, **79**, **80**, and **81** was found to be consistent with ^3^MLCT phosphorescence [91,103,109]. For complex **77**, it was evidenced as an unusual excitation-dependent variation of the emission wavelength, assigned to the presence of different molecular species in solution due to the rapid exchange between the coordinated and free terminal pyridines [109]. The incorporation of the silver ion into the structure **79** shifts the ^3^MLCT emission maximum from 557 nm to 566 nm, as well as decreasing the emission quantum yield and photostability of the macrocyclic system **82**. While the macrocyclic system **79** shows no evidence of any decomposition upon irradiation for 24 h at 405 nm, the silver-containing form easily loses the silver ion upon standing in sunlight [108]. The potential of the rhenium trimer system **79** as a metal ion transport vector was reported in [110].

Typically of d^6^ metal transition complexes, the absorption features of **83** and **84** are governed by ^1^IL and ^1^MLCT bands occurring in energy ranges of 200–340 nm and 340–500 nm, respectively. MLCT transitions attributed to Ru^II^ → π*_L_ transitions occur at lower energies (400–500 nm) compared to Re^I^-based ^1^MLCT (340–400 nm), but some mixing of Ru^II^ → π*_L_ and Re^I^ → π*_L_ is also possible [104,105]. For complex **83**, the electronic communication between Ru- and Re-based units was evidenced by the remarkable increase in nonlinear response, confirming its potential applications in optical signal processing [104,105]. Conversely to **83** which shows no noticeable changes in the solution color and the longest wavelength absorption band as the pendant pyridine converts to its pyridinium form upon acid titration, compound **84** undergoes two successive protonation–deprotonation processes upon increasing the pH from 0.40 to 10 due to the proton dissociation from the protonated imidazole group and uncoordinated pyridyl of the terpy moiety [104,105]. Excitation into any of the absorption bands of **84** resulted in a broad emission at 608 nm, attributed to the emission of the Ru-based chromophore. The attachment of {ReCl(CO)_3_(R-terpy-κ^2^N)} unit was found to induce some quenching of the emission quantum yield and lifetime, without any changes in the emission position compared to the model Ru-based chromophore. Importantly, complex **84** was demonstrated to act as a sensitive pH-induced “off–on–off” luminescence switching molecule, an efficient “turn on” emission sensor for H_2_PO_4_^−^, and a “turn off” emission sensor for F^−^ and OAc^−^. In addition, it was found to be better for cell imaging than the Ru-based chromophore [105]. The photoluminescence properties of **83** were not investigated [104].

The Re-Zn dyads (**85** and **86**) were reported in [106,107], but comprehensive photophysical studies were performed only for compound **86**. The absorption properties of the so-called hetero-Pacman compound (**86**), built as a result of the {ReCl(CO)_3_(R-terpy-κ^2^N)} fragment being covalently linked through the xanthene backbone to the porphyrin unit with the encapsulated Zn^2+^ ion, were found to be a superposition of the individual units. These include a strong (Soret) absorption centered at 423 nm and moderately intense Q-band absorption bands centered at 547 nm of the tetramesitylporphyrinato zinc unit, as well as ^1^IL (200–350) and ^1^MLCT (350–400 nm) bands of {ReCl(CO)_3_(R-terpy-κ^2^N)}. Regardless of the excitation wavelength, dyad **86** shows luminescence assigned to the fluorescence of the zinc porphyrin unit. A modest quenching effect of the attached {ReCl(CO)_3_(R-terpy-κ^2^N)} moiety on the emission quantum yield and lifetime of **86** may indicate the photoinduced electron transfer from the porphyrin S_1_ state to form a charge-separated state involving the [ReCl(CO)_3_(R-terpy-κ^2^N)]. However, no definitive spectroscopic evidence for the formation of a long-lived charge-separated state in a Re-based fragment could be found, even with the use of TA spectroscopy on the pico- and nanosecond timescales. Importantly, the Re-Zn dyad **86** exhibits enhanced photocatalytic activity in CO_2_-to-CO reduction upon excitation >450 nm relative to the corresponding Zn- and Re-based mononuclear chromophores and their 1:1 mixture.

The ferrocenyl-appended Re(I) compound (**87**) was also designed as an electrocatalyst for CO_2_ reduction, and the ferrocenyl group was introduced to extend the visible absorptivity of the dyad. The lowest energy band at 516 nm, tailing up to 650 nm, was assigned to d–d transitions in the ferrocene moiety, while the second visible absorption band was characterized as a combination of ILCT and MLCT transitions. The presence of two triplet charge-transfer excited states in energetic proximity was evidenced using TA spectroscopy [48].

## 9. Conclusions and Future Directions

Within this review, we demonstrated variations in the structural and photophysical properties of rhenium(I) carbonyl complexes with terpy-based ligands, induced by the conversion of terpy coordination mode from terpy-κ^2^N to terpy-κ^3^N, structural modifications of the terpy framework, and changes of the ancillary ligands. The Re(I) carbonyls with *meridionally*-coordinated terpys, which show the most reduced HOMO–LUMO gaps due to a significant destabilization of the HOMO level as a result of the replacement of a strongly π-accepting CO group by the weakly π-accepting pyridine of the terpy ligand, are examples of coordination compounds that show panchromatic absorption. Independent of structural modifications of the terpy framework and type of ancillary ligand, the ground- and excited-state properties of [Re(X/L)(CO)_2_(R-terpy-κ^3^N)]^0/+^ are governed by electronic transitions of a pure MLCT nature. While the emission of the halide Re(I) complexes with *meridionally*-coordinated terpys, predicted theoretically at wavelengths longer than 900 nm, has not been evidenced experimentally, the replacement of the halide ion of [ReX(CO)_2_(R-terpy-κ^3^N)] by the neutral N- and P-donor ligand induces a blue-shift of the lowest energy absorption and made it possible to obtain [ReL(CO)_2_(R-terpy-κ^3^N)]^0/+^ systems showing emission in the NIR range, where cells and tissues show negligible absorption and autofluorescence. Conversely to [Re(X/L)(CO)_2_(R-terpy-κ^3^N)]^0^/^+^, the excited-state character of [Re(X/L)(CO)_3_(R-terpy-κ^2^N)]^0^/^+^ may be switched from ^3^MLCT to ^3^IL or ^3^ILCT, which results in the formation of Re-based emitters with significantly prolonged triplet lifetimes. As demonstrated, Re(I) carbonyl complexes with a triplet excited state based on the organic ligand may be obtained by the introduction of extended π-conjugated polyaromatic hydrocarbons or strong electron-donating groups into the central pyridine of terpy. These systems were evidenced to be promising for applications involving intermolecular photoinduced energy triplet state transfer. Their usefulness as triplet photosensitizers was demonstrated in experiments concerning ^1^O_2_ generation, photocatalytic hydrogen evolution, and CO_2_-to-CO reduction. The complex [ReCl(CO)_3_(R-C_6_H_4_-terpy-κ^2^N)] with the strong electron-releasing substituent -NMe_2_ was found to be suitable for simultaneous production of CO and ^1^O_2_ in anti-tumor treatment under ultrasound irradiation, showing excellent sonocytotoxicities towards both normoxic and hypoxic cancer cells.

Regarding the promising application potential of Re(I) carbonyl complexes as luminophores, photosensitizers, and photocatalysts, the presented structure–property relationships are of high significance for better understanding and controlling the excited-state nature in these systems, and making further progress in the development of more efficient phosphorescent materials for innovative technologies, such as photodynamic therapy, time-resolved bioimaging, photocatalysis, and triplet–triplet annihilation up-conversion. Principally, triplet emitters with strong absorption in the entire visible range and sufficiently long excited-state lifetimes still face challenges. As we demonstrated in this review, such systems can be obtained via the attachment of the properly designed terpy-based ligand to the {Re(CO)_3_} and {Re(CO)_2_)} units, accompanied by suitable ancillary ligands.

## Figures and Tables

**Table 2:** The absorption and emission properties of [ReX(CO)_3_(terpy-κ^2^N)] (X = Cl, Br).

X	Medium	λ_abs_ (nm)	λ_exc_	λ_em_	τ_av_	φ_PL_ [%]	Ref.
Cl	77 K DMF–CH_2_Cl_2_ (9:1 *v*/*v*)	–	–	530	3.4 μs	–	[32]
	DMSO	320–400, 290–320	–	–	–	–	[38]
	MeCN	320–400, 250–300	360	506	–	–	[39]
	CH_2_Cl_2_	378, 295, 220	442	509	2.02 μs	0.3	[49]
	Solid	–	365	562	1.95 μs	–	
	CHCl_3_	–	393	638	4.59 ns	0.42	[46]
	MeCN	375, 323, 306	380	656	3.59 ns	<0.01	
	Solid	–	491	582	0.6 μs	–	
Br	MeCN	367, 310, 247	–	640	4.41 ns	0.03	[47]
	DMF	375, 309	–	–	–	–	[48]
	MeCN	372, 310, 247	–	–	–	–	

**Table 3:** The absorption and emission properties of [ReX(CO)_2_(terpy-κ^3^N)] (X = Cl, Br).

X	Medium	λ_abs_ (nm) (ε (10^−4^/dm^3^mol^−1^cm^−1^))	λ_exc_	λ_em_	Ref.
Cl	MeCN	689 (0.13), 567 (0.14), 466 (0.36), 398 (0.40), 321 (0.26), 280 (3.39), 271 (3.40), 239 (2.91)	–	–	[36]
	EtOH–MeOH 77 K	–	385	520	
	MeCN	671 (0.14), 460 (0.38), 397 (0.40), 320 (2.66), 280 (4.75)	–	–	[55]
Br	MeCN	690 (0.11), 547 (0.12), 446 (0.33), 395 (0.33), 330 (2.10), 323 (2.20), 278 (1.65), 241 (2.74), 214 (4.27)	–	–	[47]

## Data Availability

The datasets generated and/or analysed during the current study are available within the manuscript and Supplementary Materials.

## Supplementary Materials

The following supporting information can be downloaded at: https://www.mdpi.com/article/10.3390/molecules29071631/s1, Table S1: Selected bond lengths (Å) and angles (°) for [ReCl(CO)_3_(R-terpy-κ^2^N)]. Table S2: Selected bond lengths (Å) and angles (°) for [Re(X/L)(CO)_2_(R-terpy-κ^3^N)]^0/+^. Table S3: Selected bond lengths (Å) and angles (°) for [Re(X/L)(CO)_3_(R-terpy-κ^2^N)]^0/+^. Table S4: The absorption and emission properties of [ReX(CO)_3_(bipy)]. Table S5: The absorption and emission properties of compounds **1**–**7** (class A). Table S6: The absorption and emission properties of compounds **8**–**13** (class B). Table S7: The absorption and emission properties of compounds **14**–**29** (class C). Table S8: The absorption and emission properties of compounds **30**–**45** (class D). Table S9: The absorption and emission properties of [ReX(CO)_2_(R-terpy-κ^3^N)] (X = Cl, Br) (class E). Table S10: The absorption and emission properties of [Re(X/L)(CO)_3_(R-terpy-κ^2^N)]^0/+^ and [Re(X/L)(CO)_2_(R-terpy-κ^3^N)]^0/+^ bearing the ancillary ligands different from halide ions (class F). Table S11: Electrochemical data of [Re(X/L)(CO)_3_(R-terpy-κ^2^N)]^0/+^ and [Re(X/L)(CO)_2_(R-terpy-κ^3^N)]^0/+^. Table S12: The absorption and emission properties of higher nuclearity coordination compounds with photoactive {Re(CO)_3_(R-terpy-κ^2^N)} and {Re(CO)_2_(R-terpy-κ^3^N)} subunits (class G).
