# Supplementary material for: Structural and Photophysical Trends in Rhenium(I) Carbonyl Complexes with 2,2′:6′,2″-Terpyridines
Source: Molecules. 2024 Apr 5;29(7):1631. doi: 10.3390/molecules29071631 (PMC11013590; doi:10.3390/molecules29071631)
Supplement: Supplementary file 1 [file molecules-29-01631-s001.zip › molecules-2957087-supplementary.pdf]

SUPPORTING INFORMATION

Structural and photophysical trends in rhenium(I) carbonyl complexes with 2,2′:6′,2″-terpyridines

Joanna Palion-Gazda*, Katarzyna Choroba, Anna Maria Maroń, Ewa Malicka, Barbara Machura*

Institute of Chemistry, University of Silesia, 9 Szkolna Str., 40-006 Katowice, Poland

Table of contents

Title	Number
Selected bond lengths (Å) and angles (°) for [ReCl(CO)$_3$(R-terpy-κ^{2}N)].	**Table S1**
Selected bond lengths (Å) and angles (°) for [Re(X/L)(CO)$_2$(R-terpy-κ^{3}N)]$^{0/+}$.	**Table S2**
Selected bond lengths (Å) and angles (°) for [Re(X/L)(CO)$_3$(R-terpy-κ^{2}N)]$^{0/+}$.	**Table S3**
The absorption and emission properties of [ReX(CO)$_3$(bipy)].	**Table S4**
The absorption and emission properties of compounds **1**–**7** (class A).	**Table S5**
The absorption and emission properties of compounds **8**–**13** (class B).	**Table S6**
The absorption and emission properties of compounds **14**–**29** (class C).	**Table S7**
The absorption and emission properties of compounds **30**–**45** (class D).	**Table S8**
The absorption and emission properties of [ReX(CO)$_2$(R-terpy-κ^{3}N)] (X = Cl, Br) (class E).	**Table S9**
The absorption and emission properties of [Re(X/L)(CO)$_3$(R-terpy-κ^{2}N)]$^{0/+}$ and [Re(X/L)(CO)$_2$(R-terpy-κ^{3}N)]$^{0/+}$ bearing the ancillary ligands different from halide ions (class F).	**Table S10**
Electrochemical data of [Re(X/L)(CO)$_3$(R-terpy-κ^{2}N)]$^{0/+}$ and [Re(X/L)(CO)$_2$(R-terpy-κ^{3}N)]$^{0/+}$.	**Table S11**
The absorption and emission properties of higher nuclearity coordination compounds with photoactive {Re(CO)$_3$(R-terpy-κ^{2}N)} and {Re(CO)$_2$(R-terpy-κ^{3}N)} subunits (class G).	**Table S12**

Table S1. Selected bond lengths (Å) and angles (°) for [ReCl$(CO)_3$(R-terpy-κ^{2}N)].

	[ReCl$(CO)_3$(R-terpy-κ^{2}N)]																	
Refcode*	BOFYIF	DUZGAJ	DUZGEN	DUZGIR	KAWBIX	KUHMIM	ONEZOY	ONEZUE	ONIBAQ	ONIBEU	QIYSEZ	RISKUC	RISLEN	SUZLOQ	SUZLUW	SUZMAD	TACLIU	WELHAY
R**	Cl	CCl_3-Ph	CN-Ph	CH_3O-Ph	Nap	dimethoxy-nap	bph	Cl-Ph	Br-Ph	NH_2-Ph	TXP-H_2	pyrr-Ph	Ph	fur	thp	bthp	OH	$N(Ph)_2$
**Bond lengths**																		
Re(1)–Cl(1)	2.4944(15)	2.4973(7)	2.4965(5)	2.4990(5)	2.4848(12)	2.4986(11)	2.4847(10)	2.4647(17)	2.4596(17)	2.4996(16)	2.4898(13)	2.4904(11)	2.4862(18)	2.483(2)	2.4828(18)	2.4929(18)	2.5188(18)	2.482(2)
Re(1)–N(1)	2.210(4)	2.225(3)	2.223(2)	2.220(2)	2.218(2)	2.204(3)	2.217(3)	2.231(4)	2.227(6)	2.200(5)	2.210(3)	2.204(4)	2.223(4)	2.209(5)	2.219(4)	2.225(5)	2.217(6)	2.214(7)
Re(1)–N(2)	2.161(5)	2.162(2)	2.158(1)	2.153(1)	2.164(3)	2.165(3)	2.160(3)	2.174(6)	2.165(7)	2.173(5)	2.159(3)	2.171(3)	2.178(5)	2.157(6)	2.170(5)	2.172(4)	2.151(7)	2.185(7)
Re(1)–C(1)	1.902(6)	1.899(3)	1.904(2)	1.902(2)	1.948(5)	1.895(5)	1.919(5)	1.969(8)	1.949(9)	1.884(8)	1.941(5)	1.889(4)	1.883(8)	1.945(11)	1.898(7)	1.918(9)	1.909(8)	1.878(10)
Re(1)–C(2)	1.917(5)	1.914(3)	1.912(2)	1.914(2)	1.913(3)	1.895(6)	1.900(5)	1.897(8)	1.893(8)	1.921(8)	1.907(5)	1.895(8)	1.887(6)	1.898(8)	1.887(7)	1.912(8)	1.904(9)	1.885(10)
Re(1)–C(3)	1943(6)	1.936(3)	1.938(2)	1.931(2)	1.921(5)	1.915(4)	1.905(5)	1.928(8)	1.918(9)	1.925(8)	1.926(5)	1.915(5)	1.942(8)	1.925(10)	1.920(8)	1.932(8)	1.929(10)	1.921(10)
C(1)–O(1)	1.162(8)	1.159(4)	1.153(3)	1.149(2)	1.077(7)	1.141(6)	1.222(6)	1.065(10)	1.063(11)	1.166(10)	1.127(6)	1.159(6)	1.157(9)	1.101(14)	1.148(10)	1.123(12)	1.148(9)	1.878(10)
C(2)–O(2)	1.141(6)	1.151(4)	1.149(3)	1.147(3)	1.144(4)	1.150(8)	1.151(6)	1.158(10)	1.161(10)	1.130(12)	1.162(6)	1.157(10)	1.162(7)	1.149(10)	1.152(8)	1.135(10)	1.157(11)	1.885(10)
C(3)–O(3)	1.136(7)	1.143(3)	1.146(2)	1.150(2)	1.149(6)	1.149(6)	1.153(6)	1.157(10)	1.164(12)	1.160(9)	1.144(6)	1.159(7)	1.136(10)	1.141(12)	1.154(9)	1.140(9)	1.162(12)	1.921(10)
**Bond angles**																		
N(1)–Re(1)–Cl(1)	81.42(13)	80.83(6)	80.55(4)	80.85(4)	80.83(8)	82.48(8)	82.11(8)	85.41(12)	85.12(13)	81.13(13)	86.42(10)	81.60(8)	83.16(14)	83.35(16)	83.62(14)	80.93(13)	82.35(16)	82.14(18)
N(2)–Re(1)–Cl(1)	83.59(13)	83.73(7)	84.17(4)	86.32(4)	85.27(8)	84.38(8)	85.52(9)	84.62(13)	84.45(15)	83.71(14)	80.34(10)	83.84(8)	84.09(14)	83.10(17)	83.28(14)	86.25(14)	82.93(17)	84.41(17)
N(1)–Re(1)–N(2)	74.54(15)	74.56(8)	74.48(5)	74.52(6)	74.38(9)	74.87(11)	74.61(12)	75.0(2)	75.0(2)	74.0(2)	75.30(12)	74.21(14)	74.59(16)	74.74(19)	74.44(16)	75.23(17)	74.9(3)	74.8(2)
C(1)–Re(1)–Cl(1)	175.74(17)	178.16(10)	177.80(6)	177.08(5)	177.28(13)	178.96(17)	178.67(15)	175.2(3)	175.4(3)	177.8(2)	175.54(13)	178.08(18)	179.44(19)	179.0(2)	178.4(2)	178.8(2)	178.2(3)	178.2(3)
C(1)–Re(1)–N(1)	96.57(19)	99.85(12)	100.02(7)	97.95(7)	96.99(13)	98.44(18)	96.56(16)	92.7(2)	92.5(3)	98.1(3)	89.18(15)	96.6(2)	96.3(2)	96.1(3)	96.2(2)	98.4(3)	96.0(3)	97.1(4)
C(1)–Re(1)–N(2)	92.30(20)	94.79(10)	93.93(6)	90.80(6)	92.58(16)	96.32(18)	94.02(18)	90.7(3)	91.2(3)	94.1(3)	97.85(17)	95.09(17)	95.6(3)	95.9(3)	95.1(3)	92.6(3)	97.3(3)	93.9(4)
C(2)–Re(1)–Cl(1)	92.47(19)	91.47(9)	91.73(6)	92.18(5)	91.21(14)	89.80(15)	91.10(14)	90.50(19)	90.7(2)	91.8(2)	96.04(18)	91.35(17)	93.5(2)	93.3(3)	93.5(3)	91.9(2)	94.7(2)	94.2(3)
C(2)–Re(1)–N(1)	170.3(2)	169.09(10)	168.74(6)	166.65(7)	168.36(19)	168.65(16)	168.64(16)	169.5(3)	169.8(3)	168.4(3)	170.40(17)	168.8(2)	170.3(3)	171.1(3)	171.0(2)	168.6(2)	171.3(3)	172.2(3)
C(2)–Re(1)–N(2)	97.33(19)	97.05(9)	96.72(6)	93.78(7)	96.63(15)	96.17(18)	95.86(16)	95.1(3)	95.3(3)	96.1(3)	95.92(17)	96.5(2)	96.1(2)	96.8(2)	96.8(2)	95.5(2)	96.7(3)	98.1(3)
C(2)–Re(1)–C(1)	89.0(2)	87.63(14)	87.40(8)	88.46(8)	90.6(2)	89.4(2)	90.19(19)	90.6(3)	91.0(3)	88.6(3)	88.2(2)	90.4(3)	87.0(3)	87.1(4)	86.4(3)	88.5(4)	87.1(4)	86.4(4)
C(2)–Re(1)–C(3)	86.0(2)	88.11(12)	88.57(7)	90.61(7)	87.80(18)	88.4(2)	90.8(2)	86.5(3)	86.5(4)	87.9(3)	85.68(19)	86.3(3)	87.4(3)	86.5(3)	86.8(3)	87.2(3)	86.8(4)	85.2(4)
C(3)–Re(1)–Cl(1)	93.15(18)	91.75(10)	91.99(6)	92.25(5)	91.45(17)	91.06(12)	89.58(14)	90.96(18)	91.0(2)	93.6(2)	90.87(12)	93.56(12)	91.5(2)	92.8(3)	92.4(2)	89.8(3)	90.5(2)	97.7(3)
C(3)–Re(1)–N(1)	101.85(18)	99.76(12)	99.80(7)	103.01(7)	100.80(14)	99.98(16)	101.97(16)	103.2(2)	102.9(3)	101.7(3)	103.60(15)	102.71(19)	101.8(2)	101.9(2)	101.8(2)	101.693)	101.4(3)	101.7(3)
C(3)–Re(1)–N(2)	175.44(19)	173.21(13)	173.54(8)	177.31(7)	174.53(16)	173.50(15)	174.36(16)	175.3(2)	175.1(3)	175.2(3)	171.17(15)	176.21(18)	174.6(2)	174.9(3)	174.5(2)	175.3(3)	172.8(3)	175.1(3)
C(3)–Re(1)–C(1)	90.9(2)	88.11(12)	90.01(7)	90.61(7)	90.6(2)	88.3(2)	90.8(2)	93.7(3)	93.4(4)	88.6(3)	90.87(18)	87.42(19)	88.8(3)	88.2(3)	83.2(3)	91.4(3)	89.2(3)	90.0(4)
Dihedral angle**	53.41(18)	43.48(9)	43.31(6)	47.86(6)	48.17(13)	50.96(17)	53.32(15)	61.8(2)	60.9(2)	50.0(2)	84.98(14)	63.93(18)	50.2(2)	51.8(2)	50.8(2)	53.9(3)	59.2(3)	54.3(3)
Dihedral angle****	–	27.82(10)	25.86(5)	20.96(5)	35.64(13)	33.54(15)	22.64(13)<br>37.74(15)	19.9(3)	18.9(3)	19.5(2)	39.82(14)	13.89(15)<br>3.9(2)	10.8(3)	4.0(3)	6.4(2)	15.7(2)	-	104.2(4)<br>62.4(3)

* Refcode in the Cambridge Structural Database [1]

** Ph – phenyl, Nap – napthalen, bph – biphenyl, TXP-H_2 – 5-[4-(2,7-di-tert-butyl-9,9-dimethylxanthenyl)]-10,20-bis(mesityl)porphyrin, pyrr – pyrrolidine, fur – furan, thp – thiophene, bthp – 2,2’-bithiophene.

*** Dihedral angle between the least squares planes of the central pyridine and pendant pyridyl group.

**** Dihedral angle between the least squares planes of the central pyridine and introduced R substituent.

Table S2. Selected bond lengths (Å) and angles (°) for [Re(X/L)(CO)$_2$(R-terpy-κ^{3}N)]$^{0/+}$.

	[Re(X/L)(CO)$_2$(R-terpy-κ^{3}N)]$^{0/+}$		
Refcode*	QADLEQ	QADLIU	SOTBEK
X/L	Cl	Cl	pyridine
R	N(CH$_3$)$_2$-Ph	CCl$_3$-Ph	4-pyridine
Bond lengths			
Re(1)–X(1)	2.527(2)	2.5158(18)	2.242(3)
Re(1)–N(1)	2.124(5)	2.072(4)	2.056(2)
Re(1)–N(2)	2.144(5)	2.129(4)	2.137(2)
Re(1)–N(3)	2.164(5)	2.131(4)	2.129(2)
Re(1)–C(1)	1.826(11)	1.849(10)	1.883(3)
Re(1)–C(2)	1.917(6)	1.917(6)	1.911(3)
C(1)–O(1)	1.223(12)	1.191(11)	1.171(4)
C(2)–O(2)	1.160(7)	1.154(7)	1.159(4)
Bond angles			
N(1)–Re(1)–X(1)	94.35(17)	82.98(13)	84.39(8)
N(2)–Re(1)–X(1)	95.55(18)	85.82(13)	82.78(9)
N(3)–Re(1)–X(1)	98.78(16)	86.63(12)	88.87(9)
N(1)–Re(1)–N(2)	75.4(2)	76.52(17)	76.39(6)
N(1)–Re(1)–N(3)	74.48(19)	76.47(18)	76.31(6)
N(2)–Re(1)–N(3)	147.4(3)	152.64(16)	152.08(6)
C(1)–Re(1)–X(1)	176.5(4)	177.8(2)	177.42(10)
C(1)–Re(1)–N(1)	86.4(4)	99.2(3)	97.16(11)
C(1)–Re(1)–N(2)	81.3(4)	94.5(3)	95.57(11)
C(1)–Re(1)–N(3)	84.8(4)	94.1(3)	93.49(10)
C(2)–Re(1)–X(1)	87.7(2)	89.2(2)	93.04(13)
C(2)–Re(1)–N(1)	170.2(2)	172.1(2)	177.18(12)
C(2)–Re(1)–N(2)	104.8(2)	103.5(2)	104.47(10)
C(2)–Re(1)–N(3)	101.2(2)	102.6(2)	102.53(10)
C(2)–Re(1)–C(1)	91.7(4)	88.7(3)	85.45(14)
Dihedral angle D–A	12.8(2)	18.0(2)	12.43(7)

* Refcode in the Cambridge Structural Database [1]

Table S3. Selected bond lengths (Å) and angles (°) for $[Re(X/L)(CO)_3(R\text{-terpy-}\kappa^2N)]^{0/+}$.

	$[ReX(CO)_3(R\text{-terpy-}\kappa^2N)]^{0/+}$				
Refcode*	ALAXOD	EKOBIS	NABYIA	TACLEQ	YASTEU
X/L**	N_3^-	R-triazolate	CH_3CN	Br	py
R***	mor	2-py	H	OH	4-py
Bond lengths					
Re(1)–X(1)	2.207(2)	2.174(3)	2.145(7)	2.6570(5)	2.209(2)
Re(1)–N(1)	2.207(2)	2.208(2)	2.213(6)	2.216(3)	2.213(2)
Re(1)–N(2)	2.161(3)	2.165(2)	2.163(8)	2.165(3)	2.166(2)
Re(1)–C(1)	1.908(3)	1.914(3)	1.923(9)	1.906(5)	1.937(3)
Re(1)–C(2)	1.916(3)	1.899(3)	1.922(10)	1.904(4)	1.915(3)
Re(1)–C(3)	1.925(3)	1.939(3)	1.933(9)	1.933(4)	1.931(2)
C(1)–O(1)	1.152(3)	1.153(4)	1.148(11)	1.143(6)	1.145(4)
C(2)–O(2)	1.146(4)	1.156(3)	1.143(12)	1.154(6)	1.148(3)
C(3)–O(3)	1.155(4)	1.147(3)	1.163(12)	1.143(5)	1.145(3)
Bond angles					
N(1)–Re(1)–X(1)	79.70(8)	84.45(9)	81.0(3)	84.30(9)	86.19(8)
N(2)–Re(1)–X(1)	82.54(9)	88.29(9)	82.2(3)	84.53(9)	84.17(8)
N(1)–Re(1)–N(2)	74.42(9)	74.30(8)	75.1(3)	74.95(11)	74.45(8)
C(1)–Re(1)–X(1)	178.31(11)	178.47(9)	174.7(3)	178.66(11)	176.56(10)
C(1)–Re(1)–N(1)	99.05(10)	95.9(1)	94.2(3)	95.36(16)	90.54(10)
C(1)–Re(1)–N(2)	96.03(11)	93.25(10)	94.4(3)	96.64(15)	93.98(11)
C(2)–Re(1)–X(1)	94.51(10)	90.33(11)	95.4(4)	90.92(15)	93.38(10)
C(2)–Re(1)–N(1)	170.77(11)	167.62(11)	169.1(4)	170.19(14)	171.74(9)
C(2)–Re(1)–N(2)	97.81(11)	94.37(9)	94.2(4)	96.10(14)	96.30(9)
C(2)–Re(1)–C(1)	86.58(12)	89.61(12)	88.9(4)	89.6(2)	89.70(12)
C(2)–Re(1)–C(3)	87.27(12)	87.07(11)	87.5(4)	86.33(17)	84.99(10)
C(3)–Re(1)–X(1)	92.37(11)	88.60(11)	94.0(3)	92.25(15)	91.71910)
C(3)–Re(1)–N(1)	100.07(10)	103.97(10)	103.0(3)	102.38(14)	103.26(9)
C(3)–Re(1)–N(2)	173.05(10)	176.57(12)	175.9(3)	175.98(16)	175.74(10)
C(3)–Re(1)–C(1)	88.98(12)	89.87(12)	89.3(4)	86.56(19)	90.08(12)
Dihedral angle****	56.96(9)	65.62(9)	60.3(3)	62.64(16)	79.18(9)
Dihedral angle*****	15.08(9)	9.4(1)	–	–	33.68(9)

* Refcode in the Cambridge Structural Database [1]
** R-triazolate = 4-(ethoxycarbonyl)-5-(trifluoromethyl)-1,2,3-triazol-2-ate,
*** mor – morpholine, py – pyridine,
**** Dihedral angle between the least squares planes of the central pyridine and pendant pyridyl group.
***** Dihedral angle between the least squares planes of the central pyridine and introduced R substituent.

Table S4. The absorption and emission properties of $[ReX(CO)_3(bipy)]$ (X = Cl, Br).

**[ReCl(CO)₃(bipy)]**						
**medium**	**λ_{abs}**	**λ_{exc}**	**λ_{PL}**	**τ**	**φ_{PL}/%**	**Reference**
DMF	373, 293	–	606	–	–	[2]
CH_2Cl_2	387, 294, 239	–	–	–	–	[3]
N,N-dimethylacetamide	–	–	607	–	–	
H_2O–dioxane 1:4 (v:v)	368	–	605	29 ns	0.4	[4]
H_2O–dioxane 1:4 (v:v) 77 K	–	–	536	2.9 μs	–	
MeCN	371	–	633	–	0.27	[5]
77K 2-MeTHF	–	–	–	3.17 μs	–	
DMF–MeOH	294, 317, 371	–	598	–	0.23	[6]
MeCN	–	–	611	29 ns	0.2	[7]
$CHCl_3$	–	393, 316sh, 295	622	51.0 ns	1.65	[8]
MeCN	381, 332, 290	369, 299	639	26.4 ns	0.71	
Solid	–	471	584	0.6 μs	–	
MeCN	371, 291	–	–	–	–	[9]
MeCN	384, 295	–	–	–	–	
CH_2Cl_2	390, 290	–	620	45 ns	–	[10]
EtOH	365, 315, 290	–	620	24 ns	–	
EtOH 77 K	–	–	530	2.8 μs	–	
MeCN	370	–	612	25 ns	–	[11]
CH_2Cl_2	384	–	612	50 ns	–	
DMF	373	–	610	26 ns	–	
EtOH	372	–	604	36 ns	–	
THF	388	–	614	65 ns	–	
dioxane	390	–	614	62 ns	–	
benzene	400	–	612	70 ns	–	
CH_2Cl_2	386, 320sh, 294, 256, 242	–	622	50 ns	–	[12]
77K EtOH–MeOH	–	–	550	2.78 μs	–	
EtOH	–	–	612	–	–	[13]
EtOH 175 K	–	–	529	–	–	
2-MeTHF 80 K	375	–	532	2.7 μs	2.8	[14]
THF	389	–	612	45 ns	–	
DMF	370	370	574	46.6 ns	–	[15]
CH_2Cl_2	–	–	642	39 ns	0.31	[16]
CH_2Cl_2	385	385	600	51 ns	0.5	[17]
DMF	370	–	580	–	–	[18]
DMF	500, 466, 443, 400, 373	–	–	–	–	[19]
**[ReBr(CO)₃(bipy)]**						
**medium**	**λ_{abs}**	**λ_{exc}**	**λ_{PL}**	**τ**	**φ_{PL}/%**	**Reference**
DMF	370	–	575	–	–	[18]
MeCN	376	–	610	25 ns	–	[20]
MeCN	294, 304, 317, 377	380	579	–	0.78	[21]
toluene	301, 410	–	614	90 ns	2	[22]
DMF	580, 473, 443, 400, 373	–	–	–	–	[19]
THF	392	–	–	–	–	[14]
2-MeTHF 80 K	378	–	530	3.7 μs	4.88	
DMF	–	–	610	55 ns	–	[23]
DMF	375	–	600	4.2 ns	–	[24]

Table S5. The absorption and emission properties of compounds **1**–**7** (class A).

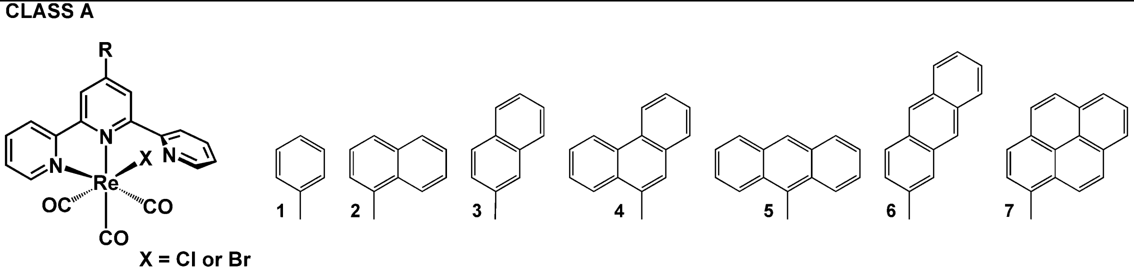

Compound	medium	λ_{abs}/nm<br>(10^4 ε/$dm^3·mol^{-1}·cm^{-1}$)	λ_{exc}	λ_{PL}	τ	τ_{av}	φ_{PL}/%	Reference
**1**^{Cl}	77K<br>(EtOH/MeOH)<br>(DMF)	–	384	543<br>570	2.28 μs (22.41 %), 5.88 μs (77.59 %)	5.07 μs		[25–28]
	solid	–	442	592	30.04 ns (65.78 %), 94.89 ns (34.22 %)	52.2 ns	1.2	
	$CHCl_3$	397 (0.28), 297 (1.60), 264 (1.82)<br>398 (0.27)	400	667	3.27 ns (71.01 %), 28.45 ns (28.99 %)	10.6 ns	0.4	
	THF	400 (0.31)	400	676	1.98 ns (61.56%), 19.52 ns (38.44%)	8.7 ns	0.2	
	DMF	385 (0.51)<br>380<br>382, 331	385	665<br>652	1.77 ns (56.75 %), 16.61 ns (43.25 %)	7.2 ns<br>1.5 ns	0.2	
	MeCN	374 (0.46), 324 (1.34), 293 (2.54), 260 (2.84), 218 (2.94)<br>375 (0.46)	380	666	2.18 ns (71.42 %), 16.01 ns (28.58 %)	6.1 ns	0.2	
**1**^{Br}	MeCN	386 (0.37), 328 (1.28sh), 266 (2.55), 218 (3.28sh)<br>380 (0.71), 266 (4.76)		527, 653	2.5 ns (65%), 38.00 (35%)	14.9 ns	0.1	[29,30]
	DMF	384 (0.93)						[30]
**2**^{Cl}	77K (BuCN)	–	420	522/558	4.93 μs (21.55 %), 316.31 μs (78.45 %)	249.2 μs		[31]
	solid	–	420	628	28.75 ns (25.45 %), 208.62 ns (74.55 %)	162.8 ns	4.8	
	$CHCl_3$	399 (1.03), 305 (4.82)	420	660	3.55 ns (86.12 %), 24.85 ns (13.88 %)	6.5 ns	7.7	
	MeCN	380 (1.14), 301 (4.25)	410	654	2.43 ns (82.81 %), 14.54 ns (17.19 %)	4.5 ns	1.9	
**3**^{Cl}	77K (BuCN)	–	420	518, 556	18.61 μs (29.81 %), 12.83 μs (70.19 %)	95.6 μs	–	
	solid	–	400	578	46.32 ns (19.72 %) 115.94 ns (80.28%)	102.2 ns	1.6	
	$CHCl_3$	404 (0.62), 313 (2.82), 269 (3.66)	450	645	3.64 ns (89.28 %) 26.89 ns (10.72 %)	6.1 ns	9.8	
	MeCN	382 (0.83), 314 (2.84), 266 (3.86)	405	663	2.82 ns (92.03 %), 18.08 ns (7.97 %)	4.0 ns	1.01	
**4**^{Cl}	77K (BuCN)	–	420	522sh, 558	1.10 μs (39.61 %), 172.52 μs (60.39 %)	104.6 μs	–	
	solid	–	370	619	17.06 ns (70.13 %), 63.34 ns (29.87 %)	13.0 ns	0.6	
	$CHCl_3$	401 (1.27), 300 (6.21), 260 (12.53), 254 (13.79)	440	665	3.35 ns (94.69 %), 20.00 ns (5.31 %)	4.2 ns	6.8	

	MeCN	376 (1.47), 297 (5.32), 257 (11.53), 251 (13.00)	410	641	2.55 ns (92.56 %), 9.43 ns (7.44%)	3.1 ns	0.6	
**5^{Cl}**	77K (BuCN)	–	430	587<br>688,761	2.35 μs (88.92 %), 13.00 μs (11.08 %)<br>12.07 ms	3.6 μs<br>12.1 ms	–	[32]
	solid	–	–	–	–	–	–	
	$CHCl_3$	416 (0.67), 386 (0.97), 368 (0.91), 351 (0.77), 308 (1.98), 258 (9.25)	445	511	0.90 ns (79.08 %), 7.43 ns (20.92 %)	4.9 ns	3.8	
	MeCN	407 (0.41), 384 (0.74), 365 (0.69), 348 (0.56), 311 (1.27), 252 (8.80)	430	627	1.35 ns (18.16 %), 5.71 ns (81.84 %)	2.3 ns	4.1	
	DMSO	406 (0.57), 388 (0.90), 368 (0.91), 349 (0.76), 328 (1.40), 316 (1.53), 260(5.33)	430	627<br>711	0.35 ns (43.93 %), 2.53 ns (33.83 %), 8.19 ns (22.24 %)<br>410 ns (59.61 %), 34.75 μs (40.39 %)	2.8 ns<br>14.30 μs	0.02	
**6^{Cl}**	77K (BuCN)	–	470	707,784	2.59 ms	2.59 ms	–	
	solid	–	–	–	–	–	–	
	$CHCl_3$	423 (0.91), 312 (3.55), 259 (4.56)	450	580	7.30 ns	7.3 ns	1.8	
	MeCN	412 (0.83), 305 (3.38), 292 (2.43), 250 (4.82)	430	608	0.57 ns (61.35 %), 9.37 ns (38.65 %)	4.0 ns	0.05	
	DMSO	421 (0.58), 360 (1.00), 312 (2.43), 262 (2.75)	430	593<br>712	0.30 ns (10.40 %), 6.38 ns (89.60 %)<br>250 ns (49.96 %), 45.13 μs (50.04 %)	5.7 ns<br>22.70 μs	1.4	
**7^{Cl}**	77K (BuCN)	–	440	627,680,756	3.22 ms (32.64 %), 6.91 ms (67.36 %)	5.74 ms	–	[31]
	solid	–	–	–	–	–	–	
	$CHCl_3$	409 (1.44), 321 (2.66), 306(2.78), 274 (3.45)	450	500<br>650, 696sh	1.72 ns (11.06 %), 5.02 ns (88.94 %)<br>4.4 μs	4.6 ns<br>4.4 μs	<0.1<br>7.6	
	MeCN	395 (1.59), 324 (2.74), 272 (3.58)	420	620	2.50 ns (82.36%). 6.68 ns (17.64 %)	3.3 ns	6.1	

Table S6. The absorption and emission properties of compounds **8–13** (class B).

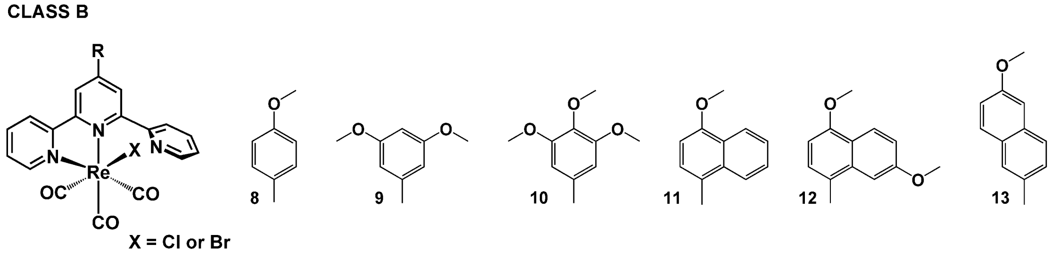

Compound	medium	λ_{abs}/nm (10^4 ε/$dm^3 \cdot mol^{-1} \cdot cm^{-1}$)	λ_{exc}	λ_{PL}	τ	τ_{av}	φ_{PL}/%	Reference
**8^{Cl}**	77K (EtOH/MeOH)<br>(DMF)	–<br>–	368, 337, 306<br>–	505, 533<br>562	6.50 μs (70.03 %), 15.62 μs (29.97 %)<br>–	9.23 μs<br>–	–<br>–	[27,33]
	solid		481, 430, 304, 265	584	586.83 ns	586.83 ns	31	
	$CHCl_3$	388 (0.83), 311 (3.35), 284 (2.87)	381, 343, 298, 267	647	4.82 ns (82.52 %), 277.80 ns (16.48%)	49.76 ns	0.2	
	MeCN	365 (1.30), 318 (3.44), 303 (3.39), 278 (3.01), 223 (4.05)	370, 337, 301, 260	661	2.95 ns	2.95 ns	0.2	
	DMF	378	–	647	–	2.30 ns	–	

**9**^{Cl}	solid	–	457	600	23.92 ns (33.42 %), 93.97 ns (66.58 %)	70.56 ns	1.4	[34]
	$CHCl_3$	395 (0.51), 305 (3.10), 276 (2.74), 264 (2.77), 240 (3.25)	393, 315, 258	660	3.08 ns	3.08 ns	0.1	
	MeCN	370 (0.63), 299 (2.85), 275 (2.81), 220 (4.52), 208 (4.64)	370, 273	672	1.99 ns (82.86 %), 4.67 ns (17.14 %)	2.45 ns	0.5	
**10**^{Cl}	solid	–	462, 380, 265	580	67.87 ns (4.28 %), 466.79 ns (95.72 %)	449.72 ns	16.3	
	$CHCl_3$	393 (0.89), 306 (3.38), 241 (3.73)	393, 317	657	3.86 ns	3.86 ns	1.3	
	MeCN	376 (1.70), 295 (4.82), 244 (5.26), 209 (8.92)	369, 285	666	2.14 ns	2.14 ns	1.1	
**11**^{Cl}	77K (EtOH/MeOH)	–	264, 316, 389	536, 568	144.42 μs (22.74%), 465.27 μs (77.26 %)	392.31 μs	–	[33,35]
	solid	–	471	574	9.54 μs (87.12 %), 2.40 μs (12.88 %)	8.62 μs	1.2	
	$CHCl_3$	384 (0.90), 306 (2.34), 241 (3.93)	384	658	4.38 ns	4.38 ns	0.4	
	MeCN	374 (4.23), 306 (9.07), 231 (18.4), 206 (23.51)	375	650	0.66 ns (16.91 %), 4.77 ns (83.09 %)	4.22 ns	0.5	
**12**^{Cl}	solid	–	477, 326	633	39.66 ns (48.33%) 137.77 ns (51.67%)	90.35 ns	0.6	[34]
	$CHCl_3$	399 (1.87), 305 (4.67), 248 (9.10)	399, 302, 260	654	4.14 ns (89.72%), 30.36 ns (10.28%)	6.84 ns	0.6	
	MeCN	387 (1.93), 305 (4.01), 248 (8.26), 220 (10.02)	387, 303, 239	656	2.88 ns (48.14%), 7.13 ns (51.86%)	5.08 ns	0.7	
**13**^{Cl}	77K (EtOH/MeOH)	–	389, 350, 330, 314	525, 565, 605	56.79 μs (38.95 %), 182.98 μs (61.05 %)	133.83 μs	–	[33]
	solid	–	489, 452, 347	631	10.33 μs (4.91 %), 51.23 μs (95.09 %)	49.22 μs	1.0	
	$CHCl_3$	392sh (1.16), 362sh (1.57), 343sh (1.68), 307sh (2.50), 293sh (2.64), 255sh (3.69)	391, 329, 300, 270	661	4.59 ns (91.41 %), 34.38 ns (8.59 %)	7.15 ns	0.2	
	MeCN	372 (1.68), 340sh (2.00), 325sh (2.17), 304sh (2.44), 278(3.23), 250(4.06), 229 (4.95), 222 (5.42)	336, 284, 240	510	4.63 ns	4.63 ns	0.3	
			384, 333, 300	661	3.26ns (87.34%), 18.43ns (12.66%)	5.18 ns		

Table S7. The absorption and emission properties of compounds **14–29** (class C).

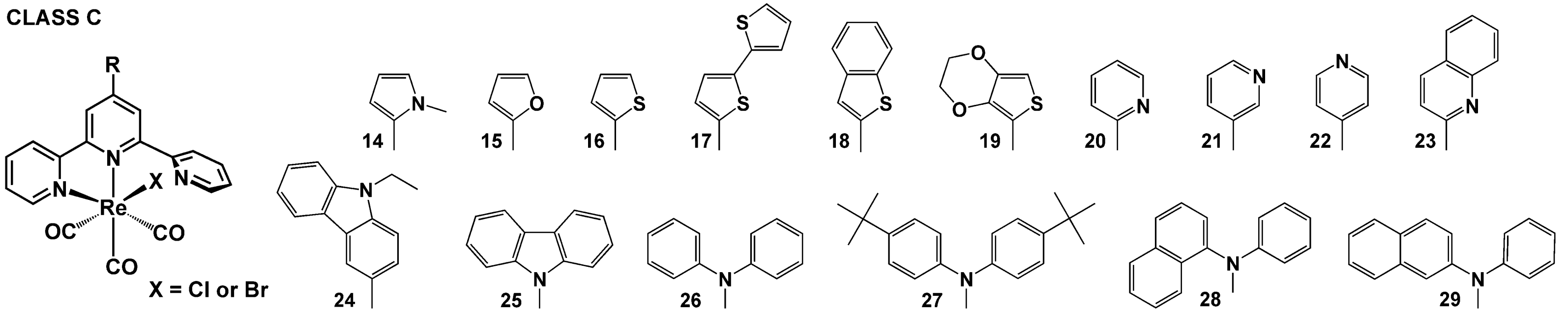

Compound	medium	λ_{abs}/nm (10^4 ε/dm^3·mol^{-1}·cm^{-1})	λ_{exc}	λ_{PL}	τ	τ_{av}	φ_{PL}/%	Reference
**14**^{Cl}	$CHCl_3$	408 (3.46), 367 (4.26), 314 (7.15), 261 (7.77)	395	656	5.70 ns	5.70 ns	0.66	[36]
	MeCN	384 (2.22), 352 (2.11), 301 (3.14), 253 (2.86), 222 (3.78)	390	646	5.11 ns	5.11 ns	0.37	
	solid	–	442	543, 567	5.95 μs (75.04%), 29.54 μs (24.96%)	11.84 μs	1.46	
	77K (EtOH/MeOH)	–	265, 312, 397	539, 576	120.89 μs (70.42%), 30.89 μs (29.58%)	94.26 μs	–	
**15**^{Cl}	$CHCl_3$	403 (2.47), 334 (9.60), 302 (8.85), 261 (7.62)	395	675	5.27 ns (91.57%), 24.75 ns (8.43%)	6.9 ns	0.20	
	MeCN	384 (0.78), 315 (4.53), 279 (5.76), 244 (6.09)	380	663	2.81 ns (53.56%), 5.55 ns (46.44%)	4.1 ns	0.20	

	solid	–	435	610	665.76 ns (86.26%), 200.00 ns (13.74%)	601.76 ns	7.15	
	77K (EtOH/MeOH)	–	307, 335, 369	528, 568	84.96 μs (76.54%), 19.25 μs (23.46%)	69.54 μs	–	
**16**^{Cl}	$CHCl_3$	407 (2.38), 333 (9.13), 292 (9.71), 260 (8.57)	395	675	4.58 ns (83.98%), 27.23 ns (16.02%)	8.0 ns	0.22	
	MeCN	381 (0.44), 321 (1.32), 258 (1.27), 194 (1.49)	380	664	3.39 ns (73.34%), 11.57 ns (26.66%)	5.6 ns	0.22	
	solid	–	458	604	430.59 ns	430.6 ns	3.50	
	77K (EtOH/MeOH)	–	307, 338, 369	525, 565	87.31 μs (72.29%), 21.78 μs (27.71%)	69.15 μs	–	
**17**^{Cl}	$CHCl_3$	428 (7.49), 327 (5.76), 260 (6.97)	390	484	2.40 ns	2.40 ns	0.15	
	MeCN	406 (1.98), 314 (1.49), 257 (1.63), 220 (2.37)	371	516	3.14 ns	3.14 ns	0.09	
	solid	–	500	689<br>758	30.10 μs (71.97%), 9.81 μs (28.03%)<br>26.68 μs (73.34%), 7.45 μs (24.66%)	24.41 μs<br>21.40 μs	1.19	
	77K (EtOH/MeOH)	–	301, 374<br>423, 447	410, 434, 467<br>661, 729	1.32 ns (68.55%), 0.32 ns (31.45%)<br>177.96 μs	1.00 ns<br>177. 96 μs	–	
**18**^{Cl}	$CHCl_3$	416 (2.58), 347 (6.38), 323 (6.94), 273 (6.43), 261 (6.58)	400	684	3.38 ns (73.23%), 6.49 ns (26.77%)	4.2 ns	0.73	
	MeCN	383 (2.2), 340 (2.68), 304 (3.26), 253 (2.74), 222 (3.87), 198 (8.0)	400	643	6.26 ns (90.66%), 1.00 ns (9.34%)	5.8 ns	0.52	
	solid	–	465	633	1.11 μs (79.42%), 20.74 μs (20.58%)	5.15 μs	7.99	
	77K (EtOH/MeOH)	–	260, 312, 341, 388	540, 583	158.48 μs (77.49%), 40.66 μs (22.51%)	131.96 μs	–	
**19**^{Cl}	$CHCl_3$	408 (2.58), 358 (4.62), 316 (6.11), 257 (1.20)	390	666	8.87 ns (88.55%), 40.64 ns (11.45%)	12.5 ns	0.58	
	MeCN	387 (0.90), 341 (1.20), 305 (1.52), 255 (1.25), 222 (1.88)	386	639	9.29 ns (85.99%), 2.67 ns (14.01%)	8.4 ns	0.32	
	solid	–	461	635	2.00 μs (51.14%), 32.69 μs (48.86%)	17.00 μs	19.47	
	77K (EtOH/MeOH)	–	313, 337, 386	540, 583	162.91 μs (70.55%), 52.40 μs (29.45%)	130.36 μs	–	
**20**^{Cl}	$CHCl_3$	404 (0.21), 318 (0.87), 287 (1.22), 263 (1.28)	401	682	1.50 ns (60.69%), 2.76 ns (39.31%)	2.00 ns	0.22	[37]
	MeCN	382 (0.31), 325 (0.91), 282 (1.66), 255 (1.77)	385	678	2.10 ns	2.10 ns	0.35	
	solid	–	480	612	59.95 ns	59.95 ns	2.74	
	77K (EtOH/MeOH)	–	370	555	4.86 μs	4.86 μs	–	
**21**^{Cl}	$CHCl_3$	405 (0.35), 307 (1.60), 276 (2.20), 261 (2.50)	402	677	2.22 ns (68.28%), 26.96 ns (31.72%)	10.07 ns	0.21	
	MeCN	382 (0.50), 310 (1.89), 273 (2.83), 256 (3.23)	380	673	1.87 ns (68.93%), 15.16 ns (31.07%)	6.00 ns	0.31	
	solid	–	493	636	61.15 ns	61.15 ns	1.76	
	77K (EtOH/MeOH)	–	367	553	3.13 μs (64.24%), 9.84 μs (35.76%)	5.53 μs	–	
**22**^{Cl}	$CHCl_3$	406 (0.40), 312 (1.62), 259 (3.27)	404	692	2.39 ns (52.32%), 0.98 ns (47.68%)	1.72 ns	0.13	
	MeCN	388 (0.44), 312 (1.52), 256 (3.65)	380	693	1.85 ns	1.85 ns	0.31	
	solid	–	461	603	52.12 ns	52.12 ns	2.91	
	77K (EtOH/MeOH)	–	365	567	4.10 μs (71.03%), 1.08 μs (28.97%)	3.22 μs	–	
**22**^{Br}	MeCN	388, 322, 265, 251	340	682	2.3 ns (85%), 21 ns (15%)	5.10 ns	0.01	[29]
**23**^{Cl}	$CHCl_3$	404 (0.6), 321 (2.8), 266 (3.7)	389	668	1.41 ns (62.30%); 6.92 ns (37.60%)	3.5 ns	0.98	[38]
	MeCN	385 (0.6), 318 (2.7), 266 (4.2)	390	663	2.35 ns (94.4%); 8.34 ns (5.54%)	2.7 ns	<0.01	
	solid	–	430	598	2.62 ns (5.03%); 249.02 ns (94.97%)	236.6 ns	7.74	
	77K (EtOH/MeOH)	–	380	585, 548, 508	41.23 μs (29.84%); 148.54 μs (70.16%)	116.52 μs	–	
**24**^{Cl}	$CHCl_3$	397 (2.2), 304 (3.7), 292 (4.0)	398	652	6.27 ns	6.27 ns	0.49	
	MeCN	391 (2.5), 302 (3.9), 286 (4.2), 230 (5.7)	376	631	0.43 ns (15.26%); 3.96 ns (84.74%)	3.4 ns	0.73	
	solid	–	462	588	64.74 ns (6.94%); 286.66 ns (93.06%)	271.3 ns	14.12	
	77K (EtOH/MeOH)	–	415	518, 551	12.77 μs (38.19%); 51.85 μs (61.81%)	36.93 μs	–	
**25**^{Cl}	CH_2Cl_2	224 (62.3), 277 (25.9), 397 (9.3)	459	578	1.37 μs	1.37 μs	0.3	[39]
	solid	–	365	551	1.66 μs	1.66 μs	–	
**26**^{Cl}	CH_2Cl_2	219 (31.4), 282 (25.3), 380 (8.0)	444	600	2.50 μs	2.50 μs	0.6	
	solid	–	367	554	1.97 μs	1.97 μs	–	
**27**^{Cl}	CH_2Cl_2	220 (36.6), 256 (29.4), 383 (8.6)	454	593	1.44 μs	1.44 μs	1.3	
	solid	–	367	532	1.87 μs	1.87 μs	–	
**28**^{Cl}	CH_2Cl_2	220 (45.5), 279 (26.8), 378 (6.9)	442	601	1.33 μs	1.33 μs	0.9	

	solid	–	365	546	3.04 µs	3.04 µs	–	
**29**^{Cl}	CH_2Cl_2	219 (34.6), 255 (20.9), 384 (5.2)	450	–	1.53 µs	1.53 µs	–	
	solid	–	365	551	2.94 µs	2.94 µs	–	

Table S8. The absorption and emission properties of compounds **30–45** (class D).

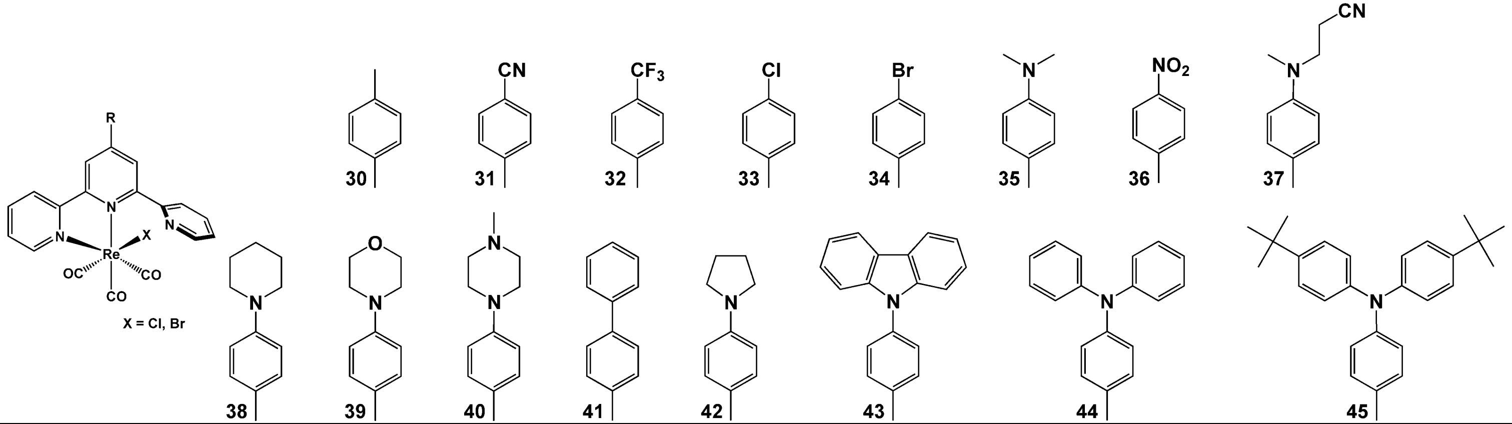

Compound	medium	λ_{abs}/nm (10^4 ε/dm^3·mol^{-1}·cm^{-1})	λ_{exc}	λ_{PL}	τ	τ_{av}	φ_{PL}/%	Reference
**30**^{Br}	MeCN	268 (2.86), 301 (2.93), 379 (0.49)	–	–	–	–	–	[30]
	DMF	307 (2.39), 382 (0.43)	–	–	–	–	–	
**31**^{Cl}	DMF	389	–	667	0.58 ns	0.58 ns	–	[27]
	DMF (77K)	–	–	585	–	–	–	
**32**^{Cl}	DMF	387	–	666	0.79 ns	0.79 ns	–	
	DMF (77K)	–	–	578	–	–	–	
**33**^{Cl}	$CHCl_3$	398.6 (4.0), 303.0 (25.1), 267.6 (23.4)	397	669	3.03 ns (91.19%), 17.99 ns (8.81%)	4.35 ns	0.13	[35]
	MeCN	385.4 (3.3), 284.9 (19.6), 264.0 (18.2), 196.6 (178.2)	376	664	2.12 (86.66%), 5.61 (13.34%)	2.56 ns	0.09	
	solid	–	475	591	493.10 ns	493.10 ns	21.00	
	77K (EtOH/MeOH)	–	264, 305, 335, 365	542	5.52 μs (66.83%), 1.90 μs (33.17%)	4.32 μs	–	
**34**^{Cl}	DMF	382	–	663	1.35 ns	1.35 ns	–	[27]
	DMF (77K)	–	–	575	–	–	–	
**34**^{Cl}	$CHCl_3$	401.2 (4.8), 304.2 (30.2), 267.9 (27.1)	396	665	2.94 (63.71%), 71.60 (36.29%)	27.86 ns	0.44	[35]
	MeCN	385.4 (5.4), 294.5 (32.5), 264.7 (30.8), 194.5 (71.8)	376	664	2.21 (87.33%), 12.91 (12.67%)	3.57 ns	0.15	
	solid	–	479	592	425.34 ns	425.34 ns	16.93	
	77K (EtOH/MeOH)	–	267, 304, 333, 365	551	5.11 μs (75.16%), 1.80 μs (24.84%)	4.29 μs	–	
**35**^{Cl}	DMF	425	–	532	380.00 ns	380.00 ns	–	[27]
	DMF (77K)	–	–	560	–	–	–	
	$CHCl_3$	429.8 (14.4), 380.2 (8.2) sh, 312.6 (14.7), 247.2 (17.1)	421	636	22.54 (84.36%), 7.11 (15.64%)	20.13 ns	1.13	[35]
	MeCN	419.2 (16.5), 354.1 (9.3), 308.7 (14.2), 246.2 (17.7), 192.2 (178.2)	452	687	0.18 (96.30%), 2.48 (3.70%)	0.26 ns	1.91	
	solid	–	494	636	0.28 μs (61.85%), 3.08 μs (38.15%)	1.35 μs	0.67	
	77K (EtOH/MeOH)	–	312, 325, 368, 444	580	263.3 μs (77.92%), 103.4 μs (22.08%)	228.0 μs	–	
	$CHCl_3$	430 (1.44)	425	637	17.4 ns (94.4%), 63.9 ns (5.6%)	20.00 ns	1.3	[25]
	THF	415 (2.69)	415	650	23.1 ns (42.54%), 64.08 ns(57.46%)	46.64 ns	4.5	

	MeCN	419 (1.65)	456	687	2.65ns (10.03%), 197.12 ns (89.97%)	177.60 ns	2.0	
	DMF	425 (2.34)	425	680	27.44 ns (1.28%), 1696.08 ns (98.72%)	1.67 µs	<0.1	
	solid	–	494	636	0.3 µs (61.8%), 3.1 µs (38.2%)	1.37 µs	0.7	
	77K (EtOH/MeOH)	–	444	580	263.0 µs (77.9%), 103.0 µs (22.1%)	227.64 µs	–	
	$CHCl_3$	430 (1.44)	425	637	17.4 ns (94.4%), 63.9 ns (5.6%)	30.55 ns	1.3	[40]
	MeCN	419 (1.65)	456	687	4.50 ns	4.50 ns	2.0	
	solid	–	494	636	0.3 µs (61.8%), 3.1 µs (38.2%)	1.37 µs	0.7	
	77K (EtOH/MeOH)	–	444	580	263 µs (77.9%), 103 µs (22.1%)	227.64 µs	–	
**35**^{Cl}	H_2O	426	405	612	9.72 ns	9.72 ns	0.052	[41]
	CH_2Cl_2	426	405	541	26.27 ns	26.27 ns	0.266	
**36**^{Cl}	H_2O	–	405	–	5.67 ns	5.67 ns	0.5	
	CH_2Cl_2	389	405	–	6.44ns	6.44ns	0.2	
**37**^{Cl}	$CHCl_3$	405 (1.72)	430	643	7.36 ns (98.0%), 30.49 ns (1.96 %)	7.81ns	2.6	[25]
	THF	406 (1.78)	445	665	5.40 ns (97.52%), 29.01 ns (2.48%)	5.99 ns	2.7	
	MeCN	405 (2.24)	415	640	11.27 (18.65%), 100.42 (76.38%)	78.80 ns	0.3	
	DMF	417 (2.26)	420	640	100.69 ns (19.67%), 696.39 ns (80.33%)	579.22 ns	0.3	
	solid	–	450	560,589,649	326.31 ns (39.61%), 1157.5 ns (60.39%)	828.26 ns	0.3	
	77K (EtOH/MeOH)	–	410	564	75.46 µs (28.46%), 211.49 µs (71.54%)	172.78 µs	–	
**38**^{Cl}	$CHCl_3$	418 (1.87)	358<br>417	510<br>634	4.10 ns<br>5.2 ns (37.7%), 23.5 ns (62.3%)	4.10 ns<br>16.60 ns	1.0<br>0.8	[40]
	MeCN	404 (1.21)	445	698	4.7 ns (52.7%), 43.4 ns (47.3%)	23.00 ns	0.8	
	solid	–	536	653	13.5 ns (39.6%), 165.7 ns (60.4%)	105.43 ns	0.4	
	77K (EtOH/MeOH)	–	435	585	4.4 µs (2.9%), 93 µs (22.1%), 277 µs (75%)	228.43 µs	–	
**39**^{Cl}	$CHCl_3$	403 (1.57)	404	650	6.2 ns (96.1%), 88.8 ns (3.9%)	40.59 ns	0.6	
	MeCN	398 (2.19)	420	661	5.9 ns (47.9%), 67.1 ns (52.0%)	37.72 ns	1.0	
	solid	–	470	571	1.4 µs (22.6%), 6.5 µs (77.4%)	5.35 µs	8.7	
	77K (EtOH/MeOH)	–	419	570	71.6 µs (27.4%), 235 µs (72.6%)	190.23 µs	–	
**40**^{Cl}	$CHCl_3$	405 (1.63)	405	645	8.10 ns	8.10ns	0.9	[25]
	THF	405 (1.75)	405	665	4.72 ns (43.81%) 7.12 ns (56.19)	6.07 ns	1.0	
	MeCN	402 (1.93)	405	665	9.00 ns (15.06%), 170.42 ns (84.94%)	146.11 ns	<0.1	
	DMF	409 (1.66)	409	650	9.51 ns (7.08%), 266.39 ns (92.92%)	248.20 ns	0.1	
	solid	–	475	613	346.71 ns (47.39%), 738.59 ns (52.61%)	552.88 ns	7.1	
	77K (EtOH/MeOH)	–	420	560	80.75 µs (32.16%), 223.81 µs (67.84%)	177.80 µs	–	
**41**^{Cl}	$CHCl_3$	–	392	664	3.67 ns (93.89%), 32.77 ns (6.11%)	5.45 ns	0.52	[35]
	MeCN	–	366	442<br>671	4.46 ns (52.20%), 1.57 ns (47.80%)<br>2.94 ns (60.41%), 1.75 ns (39.59%)	3.08 ns<br>2.47 ns	0.47	
	solid	–	462	592	494.42 ns	494.42 ns	12.46	
	77K (EtOH/MeOH)	–	263, 306, 363	516, 550	88.51 µs (66.94%), 12.86 µs (33.06%)	63.50 µs	–	
**42**^{Cl}	$CHCl_3$	420.9 (2.42), 306.9 (2.83), 246.9 (3.38)	289, 329, 404	507	3.74 ns	3.74 ns	0.14	[26,42]

			265, 303, 369, 432	617	3.04 ns (20.60%) 49.78 ns (79.40%)	40.15 ns		
	MeCN	414.1 (2.51), 308.2 (2.40), 247.7 (3.05), 191.1 (8.68)	257, 315, 365, 458	702	0.079 ns	0.079 ns	0.36	
	solid	–	302, 351, 522	625	0.70 µs (44.36%), 3.54 µs (55.65%)	2.28 µs	1.29	
	EtOH-MeOH (77K)	–	319, 429	588	93.46 µs (20.55%), 279.07 µs (79.45%)	240.93 µs	–	
**43^{Cl}**	$CHCl_3$	391 (1.51), 332 (1.67), 310 (2.73), 287 (3.87), 245 (6.46)	385, 316, 287, 261	506<br>657	7.74 ns (76.77%), 2.90 ns (23.23%)<br>3.17 ns (73.15%), 7.66 ns (26.85%)	4.00 ns<br>4.38 ns	1.39	[42]
	MeCN	380 (1.52), 338 (1.63), 309 (2.34), 288 (3.63), 237 (6.67)	370, 326, 282, 254	651	0.58 ns (13.33%), 2.60 ns (86.67%)	2.33 ns	0.58	
	solid	–	486	596	332.71 ns	332.71 ns	15.42	
	77K (EtOH/MeOH)	–	400, 322, 289, 278, 240	513, 540	9.87 µs (41.66%), 38.36 µs (58.34%)	26.49 µs	–	
**44^{Cl}**	$CHCl_3$	438 (1.57)	447	628	9.1 ns (53.3%), 252.2 ns (46.7%)	122.63 ns	0.8	[40]
	MeCN	420 (1.94)	456	695	14.6 ns	14.6 ns	0.4	
	solid	–	515	604	0.90 µs (30.6%), 3.90 µs (69.4%)	2.98 µs	1.3	
	77K (EtOH/MeOH)	–	453	569, 601sh	111 µs (31.9%), 301 µs (68.1%)	240.39 µs	–	
**45^{Cl}**	$CHCl_3$	449 (2.05), 371 (0.94), 326 (1.48), 300 (2.50)	452, 373, 301, 266	613	87.2 ns (43.0%), 19.3 ns (31.3%), 5.1 ns (25.7%)	44.85 ns	1.26	[8]
	MeCN	429 (3.18), 359 (1.33), 319 (2.59), 295 (3.36)	437, 323, 296	743	151.6 ns (97.1%), 9.1 ns (2.9%)	147.47 ns	8.92	
	solid	–	447	586	9.0 µs (24.6%), 2.6 µs (75.4%)	4.17 µs	10.08	
	77K (EtOH/MeOH)	–	465, 372, 328, 296, 260	581, 626 (sh)	323.3 µs (88.0%), 79.0 µs (12.0%)	293.98 µs	–	

Table S9. The absorption and emission properties of [ReX(CO)$_2$(R-terpy-κ^{3}N)] (X = Cl, Br) (class E).

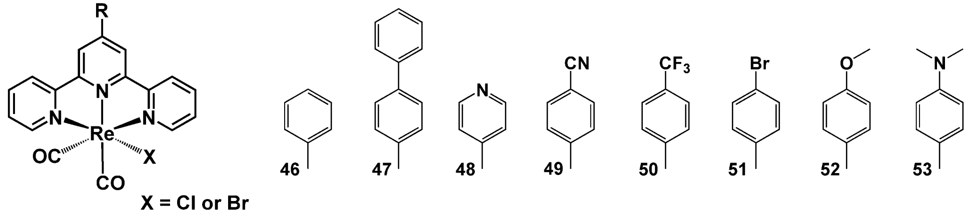

Compound	medium	λ$_{abs}$/nm (10^4 ε/dm^3·mol^{-1}·cm^{-1})	λ$_{exc}$	λ$_{PL}$	τ	τ$_{av}$	φ$_{PL}$/%	Reference
**46^{Cl}**	DMF	721, 575, 479, 407						[28]
**46^{Br}**	MeCN	700 (0.12), 569sh (0.13), 473 (0.60), 398 (0.46), 334 (2.25, sh), 323 (2.48), 286 (4.14), 239 (2.38, sh)	–	ND	ND	ND	ND	[29]
**47^{Br}**	MeCN	705 (0.14), 475 (0.88), 401 (0.58), 328 (4.66), 287 (3.76), 234 (3.24)	–	ND	ND	ND	ND	
**48^{Br}**	MeCN	721 (0.06), 481 (0.35), 402 (0.28), 328 (1.35), 283 (2.39), 227 (2.46, sh)	–	ND	ND	ND	ND	
	MeCN	~ 720, 490 (0.30), 410 (0.15), 335 (2.00), 280 (3.50),	–	ND	ND	ND	ND	[43]
**49^{Cl}**	DMF	~ 760 (0.25), 485 (0.75), 415 (0.65), 340 (2.75)	–	ND	ND	ND	ND	[28]
**50^{Cl}**	DMF	~ 760 (0.25), 485 (0.70), 410 (0.65), 340 (2.60)	–	ND	ND	ND	ND	
**51^{Cl}**	DMF	~ 760 (0.25), 485 (0.70), 410 (0.65), 320 (2.80)	–	ND	ND	ND	ND	
**52^{Cl}**	DMF	~ 740 (0.25), 480 (0.70), 400 (0.65), 330 (3.50)	–	ND	ND	ND	ND	
**53^{Cl}**	DMF	~ 710 (0.25), 490 (1.75), 410 (1.75), 310 (3.50)	–	ND	ND	ND	ND	

Table S10. The absorption and emission properties of [Re(X/L)(CO)$_3$(R-terpy-κ^{2}N)]$^{0/+}$ and [Re(X/L)(CO)$_2$(R-terpy-κ^{3}N)]$^{0/+}$ bearing the ancillary ligands different from halide ions (class F).

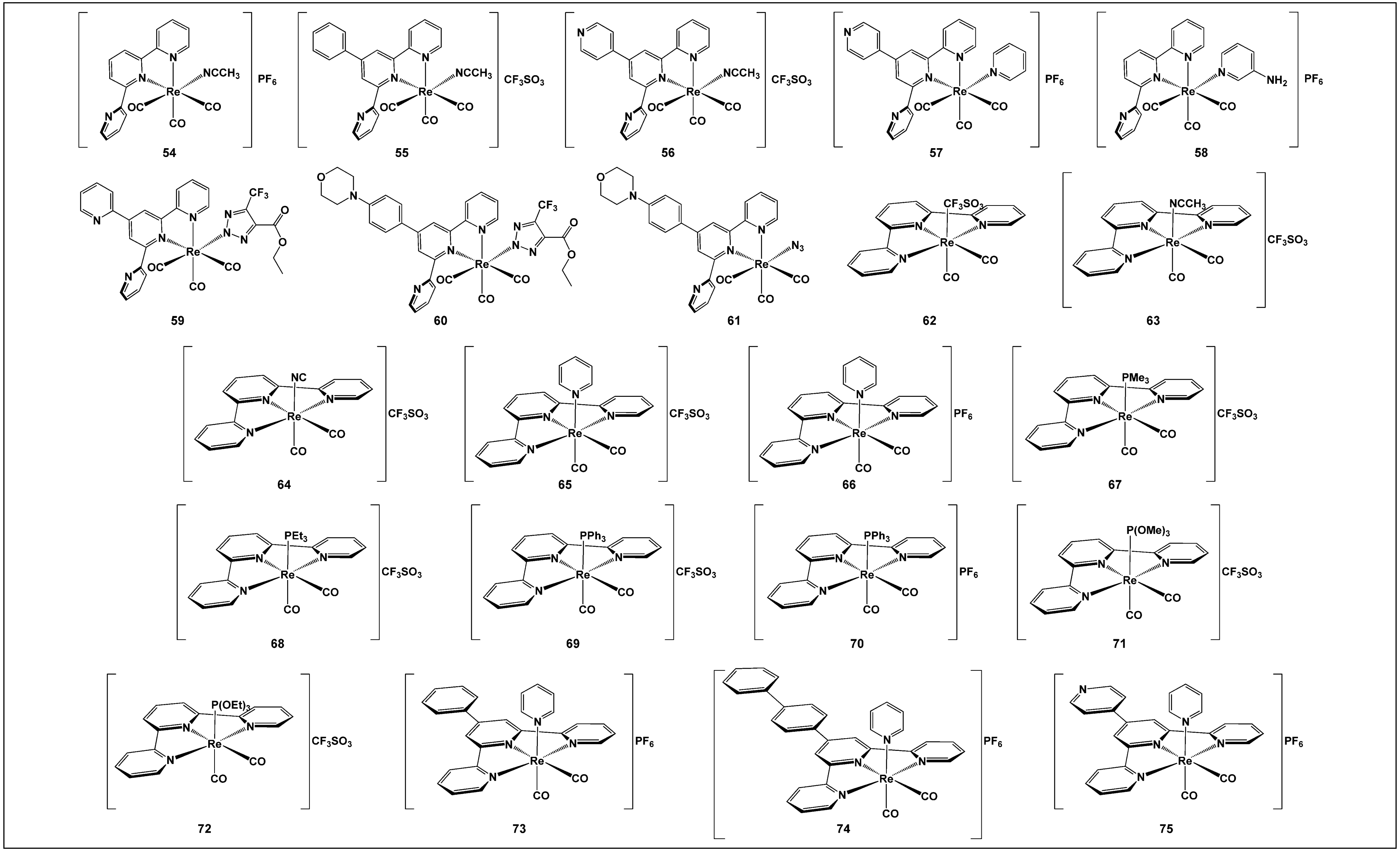

Compound	medium	λ_{abs}/nm (10^4 ε/$dm^3 \cdot mol^{-1} \cdot cm^{-1}$)	λ_{exc}	λ_{PL}	τ	τ_{av}	φ_{PL}/%	Reference
**54**	MeCN		360	510	–	–	–	[44]
**55**	MeCN	328 (1.73), 277 (2.67) 251 (2.62)	–	–	–	–	–	[45]
**56**	MeCN	330 (1.27), 279 (sh), 253 (2.81)	–	–	–	–	–	
**57**	MeCN	381 (0.40), 330 (1.31), 278 (2.59), 254 (3.42)		564	3.5 ns			[46]
**58**	MeCN	–	–	–	–	–	–	[44]
**59**	DMF<br>MeOH<br>1,4-dioxane	~ 380, 340, 280						[47]
**60**	DMSO<br>DMF<br>1,4-dioxane<br>Tol	370						[48]
**61**	DMSO<br>DMF<br>1,4-dioxane<br>Tol	413, 314<br>409<br>389<br>393						
**62**	MeCN	651 (0.17), 436 (0.64), 378 (0.55), 320 (4.71), 281 (2.38), 272 (2.45), 232 (3.95)	ND	ND	ND	ND	ND	[49]
**63**	MeCN	652 (0.16), 427 (0.54), 375 (0.46), 318 (4.16), 280 (2.39), 273 (2.05), 231 (3.81)	ND	ND	ND	ND	ND	
	MeCN	425 (0.03), 374 (0.03), 319 (0.25), 280 (0.18), 272 (0.16), 192 (0.67)	ND	ND	ND	ND	ND	[50]
**64**	MeCN	661 (0.14), 445 (0.36), 385 (0.43), 317 (2.90), 281 (1.73), 273 (1.80), 221 (3.12)	ND	ND	ND	ND	ND	[49]
**65**	MeCN	676 (0.052), 450 (0.10), 377 (0.12), 322 (0.67), 281 (0.53), 255 (0.96), 250 (0.96), 233 (1.01)	ND	ND	ND	ND	ND	
	MeCN	610 (0.029), 444 (0.069), 383 (0.86), 321 (0.61), 281 (0.41), 251	ND	ND	ND	ND	ND	[50]
**66**	MeCN	647 (0.15), 516 (0.17), 440 (0.48), 380 (0.60), 322(3.73), 281 (2.27), 274 (1.96, sh), 227 (2.95, sh)	440	870	ND	ND	0.13	[29]
**67**	MeCN	646 (0.11), 432 (0.45), 375 (0.38), 318 (3.42), 281 (1.60), 272 (1.54), 226 (2.83)	ND	ND	ND	ND	ND	[49]
**68**	MeCN	651 (0.17), 436 (0.64), 378 (0.55), 320 (4.71), 281 (2.38), 272 (2.45), 232 (3.95)	ND	ND	ND	ND	ND	
	MeCN	618 (0.011), 435 (0.031), 377, 320 (0.17), 281 (0.16), 272 (0.16)	ND	ND	ND	ND	ND	[50]
**69**	MeCN	625 (br), 421 (0.27), 371 (0.23), 319 (1.99), 273 (1.78), 228 (3.75)	ND	ND	ND	ND	ND	[49]
	MeCN	610 (0.03), 435 (0.09), 377 (0.09), 322 (0.74), 255 (3.13)	ND	ND	ND	ND	ND	[50]
	MeCN	590 (0.11), 488 (0.14), 421 (0.38), 374 (0.32), 320 (2.77), 273 (2.10)	ND	ND	ND	ND	ND	[29]
**70**	MeCN	590 (0.11), 488 (0.14), 421 (0.38), 374 (0.32), 320 (2.77), 273 (2.10)	440	800	10.2ns	10.2ns	0.76	
**71**	MeCN	600 (0.10), 408 (0.44), 367 (0.37), 312 (3.48), 279 (1.98), 272 (1.87), 230 (3.39)	ND	ND	ND	ND	ND	[49]
**72**	MeCN	575 (0.130, 530 (0.15), 481 (0.16), 411 (0.47), 368 (0.38), 314 (3.89), 5280 (1.98), 272 (1.87), 230 (3.39)	350	402 – 448<br>(77 K –125 K)	–	–	–	
**73**	MeCN	661 (0.12), 529 (0.14, sh), 448 (0.70), 382 (0.61), 323 (3.18), 285 (3.84), 240 (2.19)	440	876	ND	ND	0.13	[29]
**74**	MeCN	661 (0.13), 544 (0.14, sh), 449 (1.00), 386 (0.85sh), 325 (4.64), 285 (4.32). 235 (3.07)	440	865	ND	ND	0.11	
**75**	MeCN	678 (0.14), 539 (0.16, sh), 454 (0.82), 385 (0.72), 326 (3.38), 284 (4.45), 277(3.80, sh), 263 (2.89), 242 (2.90)	440	950	ND	ND	0.02	
	MeCN	713 (0.08), 487 (0.54), 387 (0.44), 326 (2.47), 285 (3.27), 280 (2.78), 252 (1.92), 237 (1.85)	ND	ND	ND	ND	ND	[46]

Table S11. Electrochemical data of $[Re(X/L)(CO)_3(R\text{-terpy-}\kappa^2N)]^{0/+}$ and $[Re(X/L)(CO)_2(R\text{-terpy-}\kappa^3N)]^{0/+}$.

**Compound**	**medium**	$E_{red, peak}$ [V] (ΔE [mV])	$E_{ox, peak}$ [V] (ΔE [mV])	$E_{red, onset}$ [V]	$E_{ox, onset}$ [V]	IP[a] [eV]	EA[b] [eV]	E_g[c] [eV]	**Reference**
**[ReCl(CO)$_3$(terpy-κ^{2}N)]**	CH_2Cl_2			–1.71, –2.15 (–1.79, –2.10)[d]	0.60, 0.85, 1.03 (0.57, 0.74, 0.87)[d]	–5.71	-3.39	2.31	[8]
	MeCN	–1.71	0.79						[44]
	MeCN	–1.40	1.19						[51]
**[ReBr(CO)$_3$(terpy-κ^{2}N)]**	DMF	–1.30 (77)	1.31 (irr)						[29]
	MeCN	–1.75[e], –2.13[f]	~ 2.15, ~ 0.4						[30]
**1^{Cl}**	MeCN	-1,73, -2,04	0,78, 0,91, 1,07	–1.61	0.68	–5,78	–3,49	2,29	[26]
	DMF	−1.73	The oxidation was outside of the electrochemical window of experiment (Eox°′> 0.5 V vs Fc+/Fc).	–	–	–	–	–	[27]
**1^{Br}**	DMF	–1.25 (63), –1.74 (irr)	1.23 (irr)	–	–	–	–	–	[29]
	MeCN	–1.70[e] [a] Mid-point potential for the 1st quasi-reversible reduction –2.04[f] [b] Peak potential for the irreversible 2nd reduction.	~ 2.15, ~ 0.4	–	–	–	–	–	[30]
**2^{Cl}**	CH_2Cl_2	–	–,	–1.67, –2,04 (–1.69, –2.01)[d]	0.73, 0.95 (0.71, 0.98)[d]	–5.83	–3.43	2.40	[31]
**3^{Cl}**	CH_2Cl_2	–	–	–1.63, –1.96 (–1.64, –1.96)[d]	0.67, 0.86, 1.02 (0.65, 0.84, 1.02)[d]	–5.77	–3.47	2.30	
**4^{Cl}**	CH_2Cl_2	–	–	–1.67, –2,03 (–1.65, –2.00)[d]	0.66, 1.03 (0.64, 1.02)[d]	–5.76	–3.43	2.33	
**5^{Cl}**	CH_2Cl_2	–	–	–1.76	0.67	–5.77	–3.34	2.43	[32]
**6^{Cl}**	CH_2Cl_2	–	–	–1.69	0.61	–5.71	–3.41	2.30	
**7^{Cl}**	CH_2Cl_2	–	–	–1.65, –1.97 (–1.65, –1.94)[d]	0.71, 0.97 (0.71, 0.96)[d]	–5.81	–3.45	2.36	[31]
**8^{Cl}**	DMF	–1.77	The oxidation was outside of th–e electrochemical window of experiment (Eox°′> 0.5 V vs Fc+/Fc).	–	–	–	–	–	[27]
	MeCN	–1.66, –2.13, –2.40	0.86 1.15	–1.51	0.77	–5.87	–3.59	2.28	[33]
**9^{Cl}**	CH_2Cl_2	–1.80	0.71	–1.70	0.62	–5.72	–3.40	2.32	[34]

**10**^{Cl}	CH_2Cl_2	–1.99	0.58	–1.88	0.50	–5.60	–3.22	2.38	
**11**^{Cl}	MeCN	–1.64, –2.10, –2.37	0.93, 1.18, 1.35	–1.51	0.79	–5.89	–3.59	2.30	[33]
**12**^{Cl}	CH_2Cl_2	–1.82	0.82	–1.71	0.69	–5.79	-3.39	2.40	[34]
**13**^{Cl}	MeCN	–1.67, –2.10, –2.23	0.86, 1.13, 1.31	–1.52	0.78	–5.88	–3.58	2.30	[33]
**14**^{Cl}	MeCN	–1.46, –1.77	0.77, 1.22	–1.20	0.64	–5.74	–3.90	1.84	[36]
**15**^{Cl}	MeCN	–1.28, –1.71	0.80	–1.15	0.69	–5.79	–3.95	1.84	
**16**^{Cl}	MeCN	-1,35	0.72, 1.07	–1.21	0.60	–5.70	–3.89	1.81	
**17**^{Cl}	MeCN	–1.37, –1.70	0.71, 1.06	–1.24	0.58	–5.68	–3.86	1.82	
**18**^{Cl}	MeCN	–1.34, –1.66	1.05	–1.20	0.80	–5.90	–3.90	2.00	
**19**^{Cl}	MeCN	–1.37, –1.73	0.78, 1.13	–1.18	0.62	–5.72	–3.92	1.80	
**20**^{Cl}	MeCN	–1.38, –1.66	1.14	–1.18	0.95	–6.05	–3.92	2.13	[37]
**21**^{Cl}	MeCN	–1.55, –1.84	0.93	–1.40	0.83	–5.93	–3.70	2.23	
**22**^{Cl}	MeCN	–1.56, –1.83	0.94	–1.41	0.78	–5.88	–3.69	2.19	
**22**^{Br}	DMF	–0.99 (79)	1.45 (irr)						[29]
**23**^{Cl}	MeCN	–1.78 (qr)<br>(–1.76)[d]	0.75<br>(0.54)	–1.65<br>(–1.59)	0.57<br>(0.40)	–5.67<br>(–5.50)	–3.45<br>(–3.51)	2.22<br>(1.99)	[38]
**24**^{Cl}	MeCN	–1.85<br>(–1.91)	0.75<br>(0.68)	–1.73<br>(–1.83)	0.65<br>(0.58)	–5.75<br>(–5.68)	–3.37<br>(–3.27)	2.38<br>(2.41)	
**25**^{Cl}	CH_2Cl_2	–	–	–	–	–	–	2.77	[39]
**26**^{Cll}	CH_2Cl_2	–	–	–	–	–	–	2.85	
**27**^{Cl}	CH_2Cl_2	–	–	–	–	–	–	2.83	
**28**^{Cl}	CH_2Cl_2	–	–	–	–	–	–	2.96	
**29**^{Cl}	CH_2Cl_2	–	–	–	–	–	–	2.89	
**30**^{Br}	MeCN	–1.70[e], –2.04[f]	~ 2.15, ~ 0.4						[30]
**31**^{Cl}	DMF	–1.61							[27]
**32**^{Cl}	DMF	–1.66							
**33**^{Cl}	DMF	–1.70							
	MeCN	–1.75, –2.23	0.75, 1.12	–1.63	0.59	–5.69	–3.47	2.22	[35]
**34**^{Br}	MeCN	–1.69[e], –2.03[f]	~ 2.15, ~ 0.4						[30]
**34**^{Cl}	DMF	–1.70							[27]
	MeCN	–1.70	0.77, 1.31	–1.50	0.66	–5.76	–3.60	2.16	[35]
**35**^{Cl}	DMF	–1.81							[27]
	MeCN	–1.76[d]	0.54[d]	–1.68	0.48	–5.58	–3.42	2.16	[40]
**38**^{Cl}	MeCN	–1.74[d]	0.52, 0.63, 0.99[d]	–1.61	0.42	–5.52	–3.49	2.03	
**39**^{Cl}	MeCN	–1.75	0.64, 0.87[d]	–1.65	0.57	–5.67	–3.45	2.22	
**41**^{Br}	DMF	–1.25 (60), –1.70 (irr)	1.19 (irr)						[29]
**41**^{Cl}	MeCN	–1.76	0.76	–1.63	0.60	–5.70	–3.47	2.23	[35]
**42**^{Cl}	MeCN	–1.84, –2.07, –2.23	0.58, 0.81	–1.67	0.46	–5.56	–3.43	2.13	[26]
**43**^{Cl}	MeCN	–1.78, –2.04	0.71 .0.86	–1.65	0.59	–5.69	–3.45	2.24	[42]
**44**^{Cl}	MeCN	–1.72	0.66	–1.66	0.57	–5.67	–3.44	2.23	[40]
**45**^{Cl}	MeCN			–1.70(qr), –2.03<br>(–1.74, –2.08)[d]	0.47(qr), 0.74<br>(0.40, 0.68)[d]	–5.57	–3.40	2.17	[8]
**[ReCl(CO)$_2$(terpy-κ^3N)]**	MeCN	–1.17 (iir), –1.34 (irr)	0.48 (65), 1.22 (irr)						[49]
**[ReBr(CO)$_2$(terpy-κ^3N)]**	DMF	–1.31 (qr)	0.55 (52)						[29]
**46**^{Br}	DMF	–1.27 (qr)	0.55 (63)						
**46**^{Cl}	DMF	–1.65	0.05						[28]
**47**^{Br}	DMF	–1.26 (irr)	0.50 (76), 1.26 (irr)						[29]

**48**^{Br}	DMF	−0.89 (95)*, −1.23 (irr) *affected by the addition of ferrocene	0.56 (48)						
**49**^{Cl}	DMF	–1.58	0.09						[28]
**50**^{Cl}	DMF	–1.60	0.07						
**51**^{Cl}	DMF	–1.62	0.06						
**52**^{Cl}	DMF	–1.67	0.05						
**53**^{Cl}	DMF	–1.71	0.03						
**54**	MeCN	–1.60, –1.80(irr)							[44]
**55**	MeCN	–1,15, –1.36 (irr), -1.55 (irr)	1.41 (irr)						[45]
**56**	MeCN	–1.11, –1.28 (irr), -1.60 (irr)	1.41 (irr)						
**57**	DMF	–0.92, –1.43 (qr)	1.50 (irr)						[46]
**58**	MeCN	–1.54	1.13 (irr)						[44]
**62**	MeCN	–1.19 (irr), –1.35(irr)	0.84 (irr)						[49]
**63**	MeCN	–1.21 (irr)	0.80 (irr), 1.59 (irr)						
**64**	MeCN	–0.90 (irr), –2.12 (irr)	0.67 (irr), 1.25 (irr)						
**65**	MeCN	–1.21 (irr)	0.86 (irr), 1.35 (irr)						
**66**	DMF	–1.14 (qr), –1.38 (irr)	0.82 (91)						[29]
**67**	MeCN	–1.21, –1.64 (irr)	0.84						[49]
**68**	MeCN	–1.21, –1.64 (irr)	0.84						
**69**	MeCN	–1.19 (irr)	0.94 (irr), 1.31 (irr)						
**70**	DMF	–1.11 (80)	0.95 (89)						[29]
**71**	MeCN	–1.24, –1.50 (irr)	0.92, 1.30 (irr)						[49]
**72**	MeCN	–1.23, –1.5 (irr)	0.92, 1.30 (irr)						
**73**	DMF	–1.11 (qr), –1.35 (irr)	0.82 (63)						[29]
**74**	DMF	–1.11 (qr), –1.29 (irr)	0.81 (69)						
**75**	DMF	-0.96 (qr), –1.27	0.83 (77)						
	DMF	–0.94 (qr), –1.25 (qr), –1.77 (qr)	0.86 (qr)						[46]

[a] IP = −5.1-$E_{ox\ (onset)}$, [b] EA = −5.1-$E_{red\ (onset)}$,, [c] Eg = $E_{ox\ (onset)}$ − $E_{red\ (onset)}$, [d]determined by DPV measurments, [e]Mid-point potential for the 1st quasi-reversible reduction, [f]Peak potential for the irreversible 2nd reduction.

Table S12. The absorption and emission properties of higher nuclearity coordination compounds with photoactive $\{Re(CO)_3(R\text{-terpy-}\kappa^2N)\}$ and $\{Re(CO)_2(R\text{-terpy-}\kappa^3N)\}$ subunits.

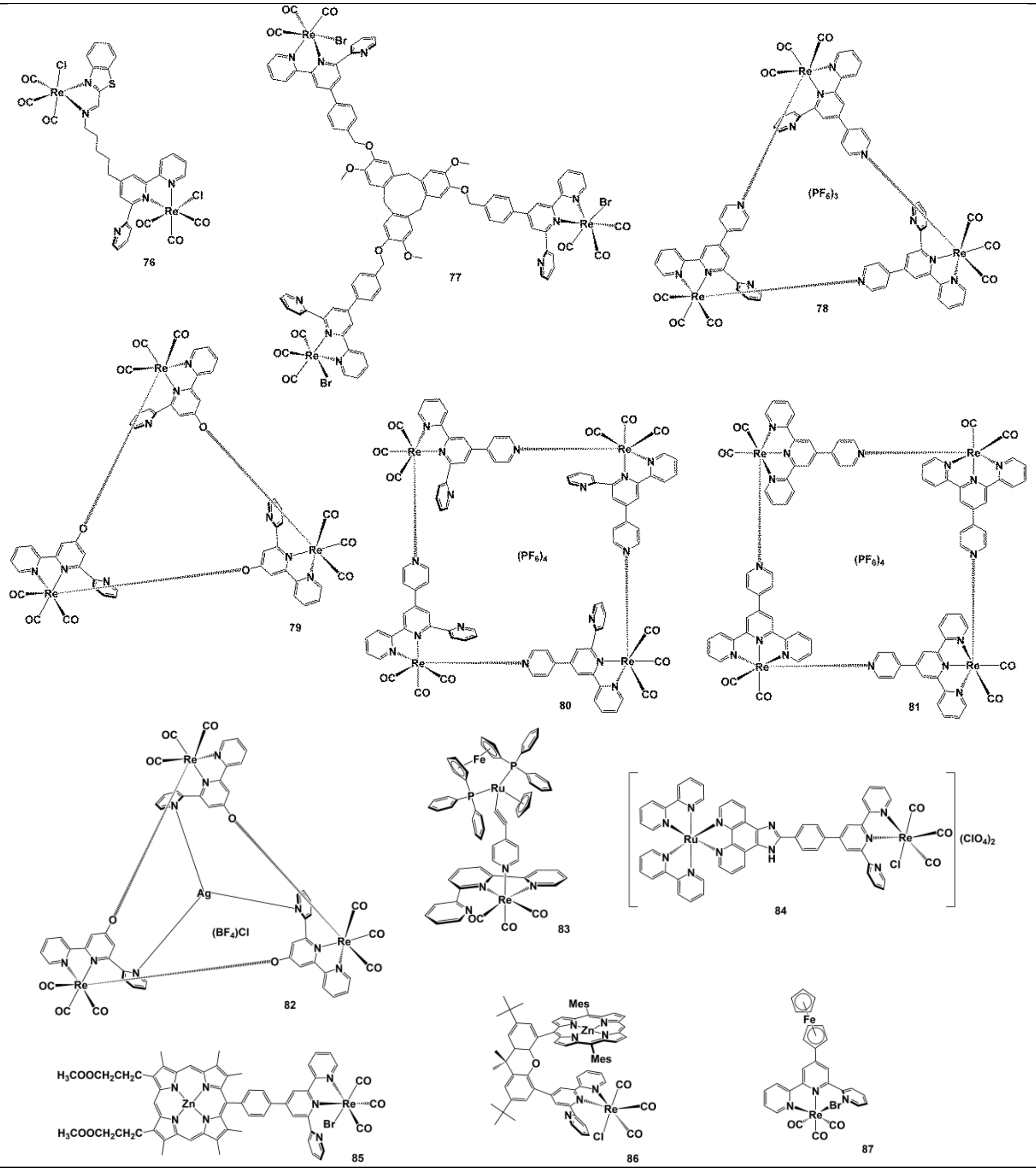

Compound	medium	λ_{abs}/nm (10^4 ε/dm^3·mol^{-1}·cm^{-1})	λ_{exc}	λ_{PL}	τ	τ_{av}	φ_{PL}/%	Reference
**76**	MeOH	250 (5.0549), 317 (2.9895), 336 (2.1075), 428 (0.7257)	-	-	-	-	-	[52]
	CH_3CN	-	428	650	-	-	-	
**77**	DMSO	280, 310, 330, 360	350–450	460–510	0.9 ns (21.5%), 3.4 ns (48.5%), 11.0 ns (30.0%)	5.10 ns	-	[53]
**78**	CH_3CN	252 (6.53), 278 (4.42), 331 (3.04), 381 (1.59)	-	546	3.6 ns	3.60 ns	-	[46]
**79**	CH_3CN	352 (1.49)	365	557	22 ns	22 ns	0.19	
**80**	CH_3CN	252 (9.96), 278 (6.79), 334 (4.85), 381 (2.53)	-	563	3.4 ns	3.40 ns	-	
**81**	CH_3CN	237 (7.03), 252 (6.82), 280 (8.10), 285 (9.06), 327 (7.37), 387 (1.83), 466 (2.57), 712 (0.17)	-	980	ND	ND	-	
**82**	CH_3CN	348 (1.33)	361	566	20 ns	20 ns	0.15	[54]
**83**	CH_2Cl_2	407 (2.3)	-	-	-	-	-	[55]
**84**	CH_3CN	285 (12.5), 337 (5.07), 423 (2.76), 455 (2.86)	460	608	21.3 ns (46%), 133.8 ns (54%)	82.00 ns	2.23	[56]
**85**	-	-	-	-	-	-	-	[57]
**86**	CH_2Cl_2	305 (5.0), 418 (41.9), 544 (2.2)	372	588, 638	2.23 ns	2.23 ns	2.20	[58]
**87**	DMF	311 (4.2), 370 (1.1), 516 (0.56)	-	-	-	-	-	[30]
	CH_3CN	256 (2.3), 310 (2.2), 367 (0.50), 516 (0.24)	-	-	-	-	-	

References:

1. Groom, C.R.; Bruno, I.J.; Lightfoot, M.P.; Ward, S.C. The Cambridge Structural Database. *Acta Crystallogr. Sect. B Struct. Sci. Cryst. Eng. Mater.* **2016**, *72*, 171–179, doi:10.1107/S2052520616003954.
2. Whang, D.R.; Apaydin, D.H.; Park, S.Y.; Sariciftci, N.S. An Electron-Reservoir Re(I) Complex for Enhanced Efficiency for Reduction of CO2 to CO. *J. Catal.* **2018**, *363*, 191–196, doi:10.1016/j.jcat.2018.04.028.
3. Stange, A.F.; Tokura, S.; Kira, M. Ruthenium and Rhenium Complexes with Silyl-Substituted Bipyridyl Ligands. *J. Organomet. Chem.* **2000**, *612*, 117–124, doi:10.1016/S0022-328X(00)00429-0.
4. Costa, I.; Montalti, M.; Pallavicini, P.; Perotti, A.; Prodi, L.; Zaccheroni, N. Absorption and Luminescence as a Function of pH for Carboxylic Acid-Functionalized ReI Tricarbonyls. *J. Organomet. Chem.* **2000**, *593–594*, 267–273, doi:10.1016/S0022-328X(99)00541-0.
5. Obata, M.; Kitamura, A.; Mori, A.; Kameyama, C.; Czaplewska, J.A.; Tanaka, R.; Kinoshita, I.; Kusumoto, T.; Hashimoto, H.; Harada, M.; et al. Syntheses, Structural Characterization and Photophysical Properties of 4-(2-Pyridyl)-1,2,3-Triazole Rhenium(I) Complexes. *Dalton Trans.* **2008**, 3292–3300, doi:10.1039/B718538C.
6. Boulay, A.; Artigau, M.; Coulais, Y.; Picard, C.; Mestre-Voegtlé, B.; Benoist, E. First Dinuclear Re/Tc Complex as a Potential Bimodal Optical/SPECT Molecular Imaging Agent. *Dalton Trans.* **2011**, *40*, 6206–6209, doi:10.1039/C0DT01397H.
7. Ramos, L.D.; da Cruz, H.M.; Morelli Frin, K.P. Photophysical Properties of Rhenium(i) Complexes and Photosensitized Generation of Singlet Oxygen. *Photochem. Photobiol. Sci.* **2017**, *16*, 459–466, doi:10.1039/c6pp00364h.
8. Maroń, A.M.; Szlapa-Kula, A.; Matussek, M.; Kruszynski, R.; Siwy, M.; Janeczek, H.; Grzelak, J.; Maćkowski, S.; Schab-Balcerzak, E.; Machura, B. Photoluminescence Enhancement of Re(I) Carbonyl Complexes Bearing D–A and D–π–A Ligands. *Dalton Trans.* **2020**, *49*, 4441–4453, doi:10.1039/C9DT04871E.
9. Cannizzo, A.; Blanco-Rodríguez, A.M.; El Nahhas, A.; Šebera, J.; Záliš, S.; Vlček, A.Jr.; Chergui, M. Femtosecond Fluorescence and Intersystem Crossing in Rhenium(I) Carbonyl−Bipyridine Complexes. *J. Am. Chem. Soc.* **2008**, *130*, 8967–8974, doi:10.1021/ja710763w.
10. Ertl, M.; Monkowius, U.; Oppelt, K.T. Unexpected Redox Chemistry of P∩N- and As∩N-Rhenium(I) Tricarbonyl Complexes in the Presence of CO2 Acting as an Acid. *Inorg. Chem.* **2023**, *62*, 17510–17521, doi:10.1021/acs.inorgchem.3c02925.
11. Kalyanasundaram, K. Luminescence and Redox Reactions of the Metal-to-Ligand Charge-Transfer Excited State of Tricarbonylchloro-(Polypyridyl)Rhenium(I) Complexes. *J. Chem. Soc. Faraday Trans. 2 Mol. Chem. Phys.* **1986**, *82*, 2401–2415, doi:10.1039/F29868202401.
12. Kalyanasundaram, K.; Graetzel, M.; Nazeeruddin, M.K. Luminescence and Intramolecular Energy-Transfer Processes in Isomeric Cyano-Bridged Rhenium(I)-Rhenium(I) and Rhenium(I)-Ruthenium(II)-Rhenium(I) Polypyridyl Complexes. *Inorg. Chem.* **1992**, *31*, 5243–5253, doi:10.1021/ic00051a016.
13. McKiernan, J.; Pouxviel, J.C.; Dunn, B.; Zink, J.I. Rigidochromism as a Probe of Gelation and Densification of Silicon and Mixed Aluminum-Silicon Alkoxides. *J. Phys. Chem.* **1989**, *93*, 2129–2133, doi:10.1021/j100342a081.
14. Rossenaar, B.D.; Stufkens, D.J.; Vlček, A. Halide-Dependent Change of the Lowest-Excited-State Character from MLCT to XLCT for the Complexes Re(X)(CO)3(α-Diimine) (X = Cl, Br, I; α-Diimine = Bpy, iPr-PyCa, iPr-DAB) Studied by Resonance

Raman, Time-Resolved Absorption, and Emission Spectroscopy. *Inorg. Chem.* **1996**, *35*, 2902–2909, doi:10.1021/ic9509802.

15. Suntrup, L.; Stein, F.; Klein, J.; Wilting, A.; Parlane, F.G.L.; Brown, C.M.; Fiedler, J.; Berlinguette, C.P.; Siewert, I.; Sarkar, B. Rhenium Complexes of Pyridyl-Mesoionic Carbenes: Photochemical Properties and Electrocatalytic CO2 Reduction. *Inorg. Chem.* **2020**, *59*, 4215–4227, doi:10.1021/acs.inorgchem.9b02591.
16. Thomas, K.R.J.; Lin, J.T.; Lin, H.-M.; Chang, C.-P.; Chuen, C.-H. Ruthenium and Rhenium Complexes of Fluorene-Based Bipyridine Ligands: Synthesis, Spectra, and Electrochemistry. *Organometallics* **2001**, *20*, 557–563, doi:10.1021/om000764n.
17. Werrett, M.V.; Chartrand, D.; Gale, J.D.; Hanan, G.S.; MacLellan, J.G.; Massi, M.; Muzzioli, S.; Raiteri, P.; Skelton, B.W.; Silberstein, M.; et al. Synthesis, Structural, and Photophysical Investigation of Diimine Triscarbonyl Re(I) Tetrazolato Complexes. *Inorg. Chem.* **2011**, *50*, 1229–1241, doi:10.1021/ic1015516.
18. Kurz, P.; Probst, B.; Spingler, B.; Alberto, R. Ligand Variations in [ReX(Diimine)(CO)3] Complexes: Effects on Photocatalytic CO2 Reduction. *Eur. J. Inorg. Chem.* **2006**, *2006*, 2966–2974, doi:10.1002/ejic.200600166.
19. El Nahhas, A.; Cannizzo, A.; van Mourik, F.; Blanco-Rodríguez, A.M.; Záliš, S.; Vlček, A.Jr.; Chergui, M. Ultrafast Excited-State Dynamics of [Re(L)(CO)3(Bpy)] n Complexes: Involvement of the Solvent. *J. Phys. Chem. A* **2010**, *114*, 6361–6369, doi:10.1021/jp101999m.
20. Bhuvaneswari, J.; Mareeswaran, P.M.; Anandababu, K.; Rajagopal, S. The Switching of a Rhenium(I) Complex from Turn-off to Turn-on Sensor System through Protein Binding. *RSC Adv.* **2014**, *4*, 34659–34668, doi:10.1039/C4RA05464D.
21. Blackburn, A.L.; Baker, N.C.A.; Fletcher, N.C. New Insights into Dihydrogenphosphate Recognition with Dirhenium(I) Tricarbonyl Complexes Bridged by a Thiourea Moiety. *RSC Adv.* **2014**, *4*, 18442–18452, doi:10.1039/C4RA00912F.
22. Kang, Y.; Ito, A.; Sakuda, E.; Kitamura, N. Characteristic Spectroscopic and Photophysical Properties of Tricarbonyl Rhenium(I) Complexes Having Multiple Arylborane Charge Transfer Units. *Bull. Chem. Soc. Jpn.* **2017**, *90*, 574–585, doi:10.1246/bcsj.20160398.
23. Kutal, C.; Weber, M.A.; Ferraudi, G.; Geiger, D. A Mechanistic Investigation of the Photoinduced Reduction of Carbon Dioxide Mediated by Tricarbonylbromo(2,2'-Bipyridine)Rhenium(I). *Organometallics* **1985**, *4*, 2161–2166, doi:10.1021/om00131a016.
24. Probst, B.; Rodenberg, A.; Guttentag, M.; Hamm, P.; Alberto, R. A Highly Stable Rhenium−Cobalt System for Photocatalytic H2 Production: Unraveling the Performance-Limiting Steps. *Inorg. Chem.* **2010**, *49*, 6453–6460, doi:10.1021/ic100036v.
25. Palion-Gazda, J.; Szłapa-Kula, A.; Penkala, M.; Erfurt, K.; Machura, B. Photoinduced Processes in Rhenium(I) Terpyridine Complexes Bearing Remote Amine Groups: New Insights from Transient Absorption Spectroscopy. *Molecules* **2022**, *27*, 7147, doi:10.3390/molecules27217147.
26. Klemens, T.; Świtlicka, A.; Szlapa-Kula, A.; Krompiec, S.; Lodowski, P.; Chrobok, A.; Godlewska, M.; Kotowicz, S.; Siwy, M.; Bednarczyk, K.; et al. Experimental and Computational Exploration of Photophysical and Electroluminescent Properties of Modified 2,2′:6′,2″-Terpyridine, 2,6-Di(Thiazol-2-Yl)Pyridine and 2,6-Di(Pyrazin-2-Yl)Pyridine Ligands and Their Re(I) Complexes. *Appl. Organomet. Chem.* **2018**, *32*, e4611, doi:10.1002/aoc.4611.
27. Fernández-Terán, R.; Sévery, L. Living Long and Prosperous: Productive Intraligand Charge-Transfer States from a Rhenium(I) Terpyridine Photosensitizer with Enhanced

Light Absorption. *Inorg. Chem.* **2021**, *60*, 1334–1343, doi:10.1021/acs.inorgchem.0c01939.
28. Fernández-Terán, R.J.; Sévery, L. Coordination Environment Prevents Access to Intraligand Charge-Transfer States through Remote Substitution in Rhenium(I) Terpyridinedicarbonyl Complexes. *Inorg. Chem.* **2021**, *60*, 1325–1333, doi:10.1021/acs.inorgchem.0c02914.
29. Auvray, T.; Del Secco, B.; Dubreuil, A.; Zaccheroni, N.; Hanan, G.S. In-Depth Study of the Electronic Properties of NIR-Emissive κ3N Terpyridine Rhenium(I) Dicarbonyl Complexes. *Inorg. Chem.* **2021**, *60*, 70–79, doi:10.1021/acs.inorgchem.0c02188.
30. Saha, S.; Doughty, T.; Banerjee, D.; Patel, S.K.; Mallick, D.; Iyer, E.S.S.; Roy, S.; Mitra, R. Electrocatalytic Reduction of CO2 to CO by a Series of Organometallic Re(I)-Tpy Complexes. *Dalton Trans.* **2023**, *52*, 15394–15411, doi:10.1039/D3DT00441D.
31. Szlapa-Kula, A.; Małecka, M.; Maroń, A.M.; Janeczek, H.; Siwy, M.; Schab-Balcerzak, E.; Szalkowski, M.; Maćkowski, S.; Pedzinski, T.; Erfurt, K.; et al. In-Depth Studies of Ground- and Excited-State Properties of Re(I) Carbonyl Complexes Bearing 2,2′:6′,2″-Terpyridine and 2,6-Bis(Pyrazin-2-Yl)Pyridine Coupled with π-Conjugated Aryl Chromophores. *Inorg. Chem.* **2021**, *60*, 18726–18738, doi:10.1021/acs.inorgchem.1c02151.
32. Małecka, M.; Szlapa-Kula, A.; Maroń, A.M.; Ledwon, P.; Siwy, M.; Schab-Balcerzak, E.; Sulowska, K.; Maćkowski, S.; Erfurt, K.; Machura, B. Impact of the Anthryl Linking Mode on the Photophysics and Excited-State Dynamics of Re(I) Complexes [ReCl(CO)3(4′-An-Terpy-κ2N)]. *Inorg. Chem.* **2022**, *61*, 15070–15084, doi:10.1021/acs.inorgchem.2c02160.
33. Klemens, T.; Świtlicka, A.; Machura, B.; Kula, S.; Krompiec, S.; Łaba, K.; Korzec, M.; Siwy, M.; Janeczek, H.; Schab-Balcerzak, E.; et al. A Family of Solution Processable Ligands and Their Re(I) Complexes towards Light Emitting Applications. *Dyes Pigments* **2019**, *163*, 86–101, doi:10.1016/j.dyepig.2018.11.035.
34. Małecka, M.; Machura, B.; Świtlicka, A.; Kotowicz, S.; Szafraniec-Gorol, G.; Siwy, M.; Szalkowski, M.; Maćkowski, S.; Schab-Balcerzak, E. Towards Better Understanding of Photophysical Properties of Rhenium(I) Tricarbonyl Complexes with Terpy-like Ligands. *Spectrochim. Acta. A. Mol. Biomol. Spectrosc.* **2020**, *231*, 118124, doi:10.1016/j.saa.2020.118124.
35. Klemens, T.; Świtlicka-Olszewska, A.; Machura, B.; Grucela, M.; Janeczek, H.; Schab-Balcerzak, E.; Szlapa, A.; Kula, S.; Krompiec, S.; Smolarek, K.; et al. Synthesis, Photophysical Properties and Application in Organic Light Emitting Devices of Rhenium(I) Carbonyls Incorporating Functionalized 2,2′:6′,2″-Terpyridines. *RSC Adv.* **2016**, *6*, 56335–56352, doi:10.1039/C6RA08981J.
36. Klemens, T.; Świtlicka-Olszewska, A.; Machura, B.; Grucela, M.; Schab-Balcerzak, E.; Smolarek, K.; Mackowski, S.; Szlapa, A.; Kula, S.; Krompiec, S.; et al. Rhenium(I) Terpyridine Complexes – Synthesis, Photophysical Properties and Application in Organic Light Emitting Devices. *Dalton Trans.* **2016**, *45*, 1746–1762, doi:10.1039/C5DT04093K.
37. Klemens, T.; Świtlicka, A.; Kula, S.; Siwy, M.; Łaba, K.; Grzelak, J.; Szalkowski, M.; Maćkowski, S.; Schab-Balcerzak, E.; Machura, B. The Effect of 2-, 3- and 4-Pyridyl Substituents on Photophysics of *Fac*-[ReCl(CO)3(n-Pytpy-κ2N)] Complexes: Experimental and Theoretical Insights. *J. Lumin.* **2019**, *209*, 346–356, doi:10.1016/j.jlumin.2019.01.045.
38. Choroba, K.; Kotowicz, S.; Maroń, A.; Świtlicka, A.; Szłapa-Kula, A.; Siwy, M.; Grzelak, J.; Sulowska, K.; Maćkowski, S.; Schab-Balcerzak, E.; et al. Ground- and Excited-State Properties of Re(I) Carbonyl Complexes – Effect of Triimine Ligand Core

and Appended Heteroaromatic Groups. *Dyes Pigments* **2021**, *192*, 109472, doi:10.1016/j.dyepig.2021.109472.
39. Wang, D.; Xu, Q.-L.; Zhang, S.; Li, H.-Y.; Wang, C.-C.; Li, T.-Y.; Jing, Y.-M.; Huang, W.; Zheng, Y.-X.; Accorsi, G. Synthesis and Photoluminescence Properties of Rhenium(I) Complexes Based on 2,2′:6′,2″-Terpyridine Derivatives with Hole-Transporting Units. *Dalton Trans.* **2013**, *42*, 2716–2723, doi:10.1039/C2DT32154H.
40. Klemens, T.; Świtlicka, A.; Szlapa-Kula, A.; Łapok, Ł.; Obłoza, M.; Siwy, M.; Szalkowski, M.; Maćkowski, S.; Libera, M.; Schab-Balcerzak, E.; et al. Tuning Optical Properties of Re(I) Carbonyl Complexes by Modifying Push–Pull Ligands Structure. *Organometallics* **2019**, *38*, 4206–4223, doi:10.1021/acs.organomet.9b00517.
41. Li, Y.; Lu, N.; Lin, Q.; Wang, H.; Liang, Z.; Lu, Y.; Zhang, P. Sono-ReCORMs for Synergetic Sonodynamic-Gas Therapy of Hypoxic Tumor. *Chin. Chem. Lett.* **2023**, *34*, 107653, doi:10.1016/j.cclet.2022.06.076.
42. Choroba, K.; Maroń, A.; Świtlicka, A.; Szłapa-Kula, A.; Siwy, M.; Grzelak, J.; Maćkowski, S.; Pedzinski, T.; Schab-Balcerzak, E.; Machura, B. Carbazole Effect on Ground- and Excited-State Properties of Rhenium(I) Carbonyl Complexes with Extended Terpy-like Ligands. *Dalton Trans.* **2021**, *50*, 3943–3958, doi:10.1039/D0DT04340K.
43. Laramée-Milette, B.; Lachance-Brais, C.; Hanan, G.S. Synthesis of Discrete Re(I) Di- and Tricarbonyl Assemblies Using a [4 × 1] Directional Bonding Strategy. *Dalton Trans.* **2014**, *44*, 41–45, doi:10.1039/C4DT03077J.
44. Amoroso, A.J.; Banu, A.; Coogan, M.P.; Edwards, P.G.; Hossain, G.; Malik, K.M.A. Functionalisation of Terpyridine Complexes Containing the Re(CO)3+ Moiety. *Dalton Trans.* **2010**, *39*, 6993–7003, doi:10.1039/C0DT00174K.
45. Metcalfe, C.; Spey, S.; Adams, H.; Thomas, J.A. Extended Terpyridyl and Triazine Complexes of D6-Metal Centres. *J. Chem. Soc. Dalton Trans.* **2002**, 4732–4739, doi:10.1039/B208211J.
46. Laramée-Milette, B.; Zaccheroni, N.; Palomba, F.; Hanan, G.S. Visible and Near-IR Emissions from k2N- and k3N-Terpyridine Rhenium(I) Assemblies Obtained by an [N×1] Head-to-Tail Bonding Strategy. *Chem. – Eur. J.* **2017**, *23*, 6370–6379, doi:10.1002/chem.201700077.
47. Mansour, A.M.; Radacki, K.; Shehab, O.R. Role of the Ancillary Ligand in Controlling the Lysozyme Affinity and Electronic Properties of Terpyridine Fac-Re(CO)3 Complexes. *Dalton Trans.* **2021**, *50*, 1197–1201, doi:10.1039/D0DT04140H.
48. Mansour, A.M.; Radacki, K. Terpyridine Based ReX(CO)3 Compounds (X = Br–, N3– and Triazolate): Spectroscopic and DFT Studies. *Polyhedron* **2021**, *194*, 114954, doi:10.1016/j.poly.2020.114954.
49. Frenzel, B.A.; Schumaker, J.E.; Black, D.R.; Hightower, S.E. Synthesis, Spectroscopic, Electrochemical and Computational Studies of Rhenium(I) Dicarbonyl Complexes Based on Meridionally-Coordinated 2,2′:6′,2″-Terpyridine. *Dalton Trans.* **2013**, *42*, 12440–12451, doi:10.1039/C3DT51251G.
50. Black, D.R.; Hightower, S.E. Preparation and Characterization of Rhenium(I) Dicarbonyl Complexes Based on the Meridionally-Coordinated Terpyridine Ligand. *Inorg. Chem. Commun.* **2012**, *24*, 16–19, doi:10.1016/j.inoche.2012.07.034.
51. Juris, A.; Campagna, S.; Bidd, I.; Lehn, J.M.; Ziessel, R. Synthesis and Photophysical and Electrochemical Properties of New Halotricarbonyl(Polypyridine)Rhenium(I) Complexes. *Inorg. Chem.* **1988**, *27*, 4007–4011, doi:10.1021/ic00295a022.
52. Ismail, M.B.; Booysen, I.N.; Hosten, E.; Akerman, M.P. Synthesis, Characterization and DNA Interaction Studies of Tricarbonyl Rhenium(I) Compounds Containing Terpyridine

Schiff Base Chelates. *J. Organomet. Chem.* **2017**, *833*, 1–9, doi:10.1016/j.jorganchem.2017.01.017.
53. Thorp-Greenwood, F.L.; Pritchard, V.E.; Coogan, M.P.; Hardie, M.J. Tris(Rhenium Fac-Tricarbonyl) Polypyridine Functionalized Cyclotriguaiacylene Ligands with Rich and Varied Emission. *Organometallics* **2016**, *35*, 1632–1642, doi:10.1021/acs.organomet.6b00099.
54. Coogan, M.P.; Fernández-Moreira, V.; Kariuki, B.M.; Pope, S.J.A.; Thorp-Greenwood, F.L. A Rhenium Tricarbonyl 4′-Oxo-Terpy Trimer as a Luminescent Molecular Vessel with a Removable Silver Stopper. *Angew. Chem. Int. Ed.* **2009**, *48*, 4965–4968, doi:10.1002/anie.200900981.
55. Ge, Q.; Corkery, T.C.; Humphrey, M.G.; Samoc, M.; Hor, T.S.A. Organobimetallic RuII–ReI4-Ethynylpyridyl Complexes: Structures and Non-Linear Optical Properties. *Dalton Trans.* **2009**, 6192–6200, doi:10.1039/B902800E.
56. Zheng, Z.-B.; Wu, Y.-Q.; Wang, K.-Z.; Li, F. pH Luminescence Switching, Dihydrogen Phosphate Sensing, and Cellular Uptake of a Heterobimetallic Ruthenium(II)–Rhenium(I) Complex. *Dalton Trans.* **2014**, *43*, 3273–3284, doi:10.1039/C3DT52568F.
57. Suzuki, M.; Uehara, T.; Arano, Y.; Hoshino, T.; Neya, S. Fabrications of Potential Imaging Probes Based on a β-Alkyl Substituted Porphyrin with a Terpyridine External Coordination Site. *Tetrahedron Lett.* **2011**, *52*, 7164–7167, doi:10.1016/j.tetlet.2011.10.132.
58. Lang, P.; Pfrunder, M.; Quach, G.; Braun-Cula, B.; Moore, E.G.; Schwalbe, M. Sensitized Photochemical CO2 Reduction by Hetero-Pacman Compounds Linking a ReI Tricarbonyl with a Porphyrin Unit. *Chem. – Eur. J.* **2019**, *25*, 4509–4519, doi:10.1002/chem.201806347.
